# Histone lactylation-boosted ALKBH3 potentiates tumor progression and diminished promyelocytic leukemia protein nuclear condensates by m^1^A demethylation of SP100A

**DOI:** 10.1093/nar/gkad1193

**Published:** 2023-12-20

**Authors:** Xiang Gu, Ai Zhuang, Jie Yu, Ludi Yang, Shengfang Ge, Jing Ruan, Renbing Jia, Xianqun Fan, Peiwei Chai

**Affiliations:** Department of Ophthalmology, Shanghai Key Laboratory of Orbital Diseases and Ocular Oncology, Shanghai Ninth People's Hospital, Shanghai Jiao Tong University School of Medicine, Shanghai 200011, People's Republic of China; Department of Ophthalmology, Shanghai Key Laboratory of Orbital Diseases and Ocular Oncology, Shanghai Ninth People's Hospital, Shanghai Jiao Tong University School of Medicine, Shanghai 200011, People's Republic of China; Department of Ophthalmology, Shanghai Key Laboratory of Orbital Diseases and Ocular Oncology, Shanghai Ninth People's Hospital, Shanghai Jiao Tong University School of Medicine, Shanghai 200011, People's Republic of China; Department of Ophthalmology, Shanghai Key Laboratory of Orbital Diseases and Ocular Oncology, Shanghai Ninth People's Hospital, Shanghai Jiao Tong University School of Medicine, Shanghai 200011, People's Republic of China; Department of Ophthalmology, Shanghai Key Laboratory of Orbital Diseases and Ocular Oncology, Shanghai Ninth People's Hospital, Shanghai Jiao Tong University School of Medicine, Shanghai 200011, People's Republic of China; Department of Ophthalmology, Shanghai Key Laboratory of Orbital Diseases and Ocular Oncology, Shanghai Ninth People's Hospital, Shanghai Jiao Tong University School of Medicine, Shanghai 200011, People's Republic of China; Department of Ophthalmology, Shanghai Key Laboratory of Orbital Diseases and Ocular Oncology, Shanghai Ninth People's Hospital, Shanghai Jiao Tong University School of Medicine, Shanghai 200011, People's Republic of China; Department of Ophthalmology, Shanghai Key Laboratory of Orbital Diseases and Ocular Oncology, Shanghai Ninth People's Hospital, Shanghai Jiao Tong University School of Medicine, Shanghai 200011, People's Republic of China; Department of Ophthalmology, Shanghai Key Laboratory of Orbital Diseases and Ocular Oncology, Shanghai Ninth People's Hospital, Shanghai Jiao Tong University School of Medicine, Shanghai 200011, People's Republic of China

## Abstract

Albeit N1-Methyladenosine (m^1^A) RNA modification represents an important regulator of RNA metabolism, the role of m^1^A modification in carcinogenesis remains enigmatic. Herein, we found that histone lactylation enhances ALKBH3 expression and simultaneously attenuates the formation of tumor-suppressive promyelocytic leukemia protein (PML) condensates by removing the m^1^A methylation of SP100A, promoting the malignant transformation of cancers. First, ALKBH3 is specifically upregulated in high-risk ocular melanoma due to excessive histone lactylation levels, referring to m^1^A hypomethylation status. Moreover, the multiomics analysis subsequently identified that SP100A, a core component for PML bodies, serves as a downstream candidate target for ALKBH3. Therapeutically, the silencing of ALKBH3 exhibits efficient therapeutic efficacy in melanoma both *in vitro* and *in vivo*, which could be reversed by the depletion of SP100A. Mechanistically, we found that YTHDF1 is responsible for recognition of the m^1^A methylated SP100A transcript, which increases its RNA stability and translational efficacy. Conclusively, we initially demonstrated that m^1^A modification is necessary for tumor suppressor gene expression, expanding the current understandings of dynamic m^1^A function during tumor progression. In addition, our results indicate that lactylation-driven ALKBH3 is essential for the formation of PML nuclear condensates, which bridges our knowledge of m^1^A modification, metabolic reprogramming, and phase-separation events.

## Graphical Abstract

**Graphical Abstract:**
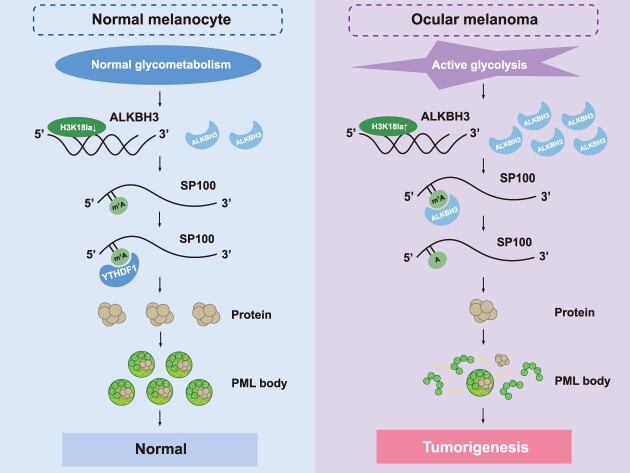

## Introduction

RNA modifications are modified or removed by a variety of enzymes, and these modifications play roles in essential molecular mechanisms (1). The precise temporal and spatial regulation of N^1^-methyladenosine (m^1^A) RNA modification is essential for epigenome homeostasis (2). Importantly, m^1^A RNA methylation blocks the Watson-Crick interface and thereby influences RNA secondary structures and protein-RNA interactions, which are observed in tRNAs, rRNAs, mRNAs and mitochondrial RNAs (3,4). Dynamic m^1^A methylation is mediated by methyltransferases (writers: TRMT6, TRMT61A, TRMT61B, and TRMT10C), demethylases (erasers: ALKBH1 and ALKBH3) and RNA-binding proteins (readers: YTHDFs) (5,6). To date, emerging evidence has demonstrated that dynamic m^1^A methylation plays vital roles in the biological processes, including gene expression, RNA stability and translational efficacy (7–9). For example, ALKBH3-mediated m^1^A demethylation is essential for Aurora A expression, which regulates ciliogenesis in mammalian cells (10).

Notably, m^1^A methylation participates in carcinogenesis of diversified cancers, including gastrointestinal cancer (11), oral squamous cell carcinoma (12), cervical cancer (13) and Hodgkin lymphoma (14). Moreover, aberrant m^1^A methylation triggers several oncogenic events, including metabolic reprogramming (8), collagen production (14) and the generation of tRNA-derived small RNAs (15). Notably, the increased expression of the m^1^A demethylase ALKBH3 is frequently observed in many cancers and accelerates malignant proliferation and invasion (9). Notably, several genome-wide screening studies have demonstrated over ∼500 salient m^1^A binding sites (16,17); however, the underlying functions of these m^1^A methylation modifications in carcinogenesis remain poorly understood.

Ocular melanoma, including conjunctival melanoma (CoM) and uveal melanoma (UM), is the most frequent and life-threatening form of ocular malignancy and is resistant to common chemotherapy. For CoM, gain-of-function mutations of *BRAF* and *NRAS* have been identified as driver oncogenic events (18). Most UM patients harbor activating mutations in *G protein subunit alpha Q (GNAQ)* and *G protein subunit alpha 11 (GNA11)* (19). Moreover, several epigenetic abnormalities are involved in the pathogenesis of ocular melanoma, including aberrant histone modifications, m^6^A modifications and 3D chromosomal conformations (20–22). For example, ALKBH5-mediated m^6^A demethylation inhibits the translation of histidine triad nucleotide-binding protein 2 (HINT2), which promotes the malignant proliferation and metastasis of ocular melanoma cells (20). However, the mechanism of m^1^A modification in ocular melanoma tumorigenesis remains enigmatic.

We thus aimed to identify the molecular mechanisms and potential clinical application of m^1^A modification targets in ocular melanoma. In this study, we revealed for the first time that global m^1^A modification is specifically decreased in ocular melanoma, which is attributed to the increased ALKBH3 expression by histone lactylation. Moreover, genome-wide proteomic analysis, transcriptome screening and m^1^A-methylated RNA immunoprecipitation sequencing (MeRIP-seq) were performed. We revealed that ALKBH3 removes the m^1^A modification on SP100A and inhibits its expression. Importantly, the silencing of ALKBH3 exhibits great therapeutic efficacy in melanoma both *in vitro* and *in vivo*, and these effects are attenuated by the depletion of SP100A. Furthermore, YTHDF1 is responsible for recognition of the m^1^A modification of SP100A, which enhances its RNA stability and translational efficacy. Overall, our results demonstrate that ALKBH3-dependent m^1^A demethylation is essential for the formation of PML nuclear bodies, thereby providing a novel therapeutic strategy in which the ‘targeted m^1^A reprogramming strategy’ is efficient for tumor therapy.

## Materials and methods

### Patients and tissue specimens

We collected 82 human ocular melanoma tissues and 28 human normal nevus tissues from the Department of Ophthalmology, Shanghai Ninth People's Hospital, Shanghai Jiao Tong University School of Medicine. The clinicopathological and prognostic information of these tissue specimens was described previously (20,21).

### Cell culture

The human UM cell lines 92.1, MUM2B and OCM1 were provided by Professor John F. Marshall (Tumor Biology Laboratory, Cancer Research UK Clinical Center, John Vane Science Centre, London, UK). The human UM cell lines MEL290, OMM2.3, and OMM1 and the human conjunctival melanoma cell lines CRMM1, CRMM2, and CM2005.1 were kind gifts from Professor Martine J. Jager (Department of Ophthalmology, Leiden University Medical Center, Leiden, The Netherlands). The human normal melanocyte cell line PIG1 was kindly provided by the Department of Ophthalmology, Peking University Third Hospital. HEK293T cells were purchased from the American Type Culture Collection (Manassas, VA, USA). All cell lines were authenticated by short tandem repeat (STR) profiling. MUM2B, OCM1 and HEK293T cells were cultured in DMEM (Gibco, USA). 92.1, MEL290, OMM2.3, OMM1 and PIG1 cells were cultured in RPMI 1640 medium (Gibco, USA), and CRMM1, CRMM2, CM2005.1 cells were cultured in Ham's F-12 K (Gibco, USA). All medium types were supplemented with 10% certified heat-inactivated fetal bovine serum (FBS), penicillin (100 U/mL), and streptomycin (100 mg/ml), and the cells were cultured at 37°C in a humidified 5% CO_2_ atmosphere.

### Dot blot assay

Total RNA was extracted using TRIzol Reagent (Invitrogen, USA), and then was quantified and diluted in 10 mM Tris-EDTA buffer. Equivalent amounts of RNA samples were loaded onto Amersham Hybond-N + membranes (GE Healthcare, USA). The membrane was UV-crosslinked, blocked with 5% milk for 1 h and incubated with an anti-m^1^A antibody (ab208196, Abcam, USA) at 4°C overnight, followed by HRP-conjugated anti-rabbit IgG (CST, USA) for 1 h at room temperature (RT), and then detected with ECL SuperSignal Western Blotting Detection Reagent (Thermo Fisher Scientific, USA).

### Immunofluorescence (IF)

Paraffin-embedded tissues were deparaffinized, rehydrated, fixed with 4% formaldehyde for 30 min, blocked with 5% normal goat serum for 1 h, and permeabilized with 0.5% Triton X-100 for 15 min. Then, they were incubated with the primary antibodies at 4°C overnight and the secondary antibodies for 1 h. Next, DAPI (Sigma-Aldrich, USA) was employed to stain nuclei for 10 min. A ZEISS Axio Scope A1 upright microscope (Germany) and a Nikon Eclipse 80i microscope (Japan) were used to acquire digital images. The following antibodies were used in this study: anti-ALKBH3 (ab251697, Abcam, USA), anti-Pan Kla (1401RM, PTM, China), anti-H3K18la (1406RM, PTM, China), anti-SP100A (NBP1-89457, Novus Biologicals, USA), and anti-PML (ab96051, Abcam, USA).

### Western blot

Cell and tissue were lysed with RIPA lysis buffer supplemented with protease and phosphatase inhibitor cocktails (NCM Biotech, China) for 30 min at 4°C and then centrifuged at 13000 × g for 30 min. Protein supernatants were separated by 7.5% (wt/vol) SDS-PAGE and transferred to PVDF membranes (Millipore Corporation, USA). After blocking with 5% milk for 1 h at RT, the membranes were incubated with the primary antibodies at 4°C overnight and then with secondary antibodies conjugated to a fluorescent tag (Invitrogen, USA). The band signals were visualized by an Odyssey Infrared Imaging System (LI-COR, USA). The following primary antibodies were used in the study: anti-ALKBH3 (ab251697, Abcam, USA), anti-LDHA (19987–1-AP, Proteintech, China), anti-LDHB (14824–1-AP, Proteintech, China), anti-SP100 (PA5-53476, ThermoFisher, USA), anti-PML (ab179466, Abcam, USA), anti-YTHDF1 (57530, CST, USA), anti-Pan Kla (1401RM, PTM, China), anti-H3K18la (1406RM, PTM, China), anti-Flag (2368S, CST, USA), anti-ACTB (3700, CST, USA) and anti-Histone H3 (4499, CST, USA). Unprocessed western blot figures were performed in Supplementary Figures S14 and S15.

### RNA isolation, reverse transcription, and real-time quantitative PCR (qPCR)

Total RNA was extracted from cultured cells using TRIzol Reagent (Invitrogen, USA), and cDNA was synthesized using PrimeScript RT Reagent Mix (Takara Bio, USA) according to the manufacturer's instructions. qPCR was performed using SYBR Green PCR Master Mix (Life Technologies, USA) and an ABI 7500 real-time PCR system (Applied Biosystems, USA). The levels of targets were normalized to those of ACTB and to those of control samples. The primers for qPCR are listed in Supplementary Table S1.

### Plasmid construction

The PGMLV-CMV-MCS-EF1-ZsGreen1-T2A-Puro, PGMLV-hU6-MCS-CMV-ZsGreen1-PGK-Puro-WPRE, ZV502 and ZV102 vectors were used in our study. ALKBH3 shRNAs, SP100A shRNA, YTHDF1 shRNA and their corresponding verified negative control sequences were amplified by PCR and inserted into the PGMLV-hU6-MCS-CMV-ZsGreen1-PGK-Puro-WPRE vectors. The SP100A ORF was generated by PCR and cloned into the PGMLV-CMV-MCS-EF1-ZsGreen1-T2A-Puro vector. The sequences of the constructed plasmids constructed are listed in Supplementary Table S1.

### Lentiviral packaging and generation of stable cell lines

A mixture of 3 μg of the indicated plasmid, 3 μg of pMD2.D plasmid, and 6 μg of PsPax plasmid was transfected into HEK239T cells by Lipofectamine 2000 (Invitrogen, USA) in Opti-MEM I Reduced Serum Medium (Gibco, USA). The medium was substituted with fresh complete medium 6 h after transfection. Forty-eight hours and 72 h after transfection, the virus-containing supernatant was collected, filtered by 0.45-mm cellulose acetate filters, and concentrated with a Lenti-X Concentrator (Takara Bio, USA). Medium comprising 25 μL/ml lentivirus and 8 ng/ml polybrene (Sigma-Aldrich, USA) was used to replace the original medium for cells seeded 24 h before transduction. After 48 h, the selection of a stable cell line was performed by incubation with 4 μg/ml puromycin (InvivoGen, USA) for 2 weeks, and a maintenance dose of 1 μg/ml puromycin was used thereafter.

### Cell proliferation assays

CCK-8 colorimetric assays were employed to assess cell proliferation capability. A total of 2000–3000 cells were seeded into 96-well plates (Corning, USA) with 100 μL medium. For 2.5 h prior to detection, 10 μL of CCK-8 solution (Dojindo, Japan) was added and the samples were incubated at 37°C. To measure the absorbance of the samples at 450 nm, a microplate reader (ELX800, BioTek, USA) was employed.

### Colony formation assay

A total of 2 ml of medium comprising 1000 cells was seeded in a 6-well plate, and the medium was replaced every 3–4 days. After 14–18 days, the colonies were stained with 0.25% crystal violet.

### Transwell assay

A 24-well Transwell system with polycarbonate filters (8 μm pores) was employed to evaluate the cell migration capability. A total of 5.0 × 10^4^ –1.0 × 10^5^ cells in medium containing 2% FBS was added to the upper compartment, and 900 μl of complete medium was placed in the lower chamber. After a 24 h incubation, the Transwell system was stained with 0.25% crystal violet. The cells migrating to the lower chamber were imaged.

### Xenograft models

The xenograft models were established in a specific pathogen-free (SPF) animal room. Four-week-old male BALB/c nude mice were anesthetized, and their scleras were preperforated with a sharp 30-gauge injection needle. A total of 2 × 10^6^ UM cells were injected into the choroid. The infected eyes were protected with ophthalmic bacitracin ointment. In vivo small animal imaging systems were employed to detect bioluminescence after 28 days. The tumors were fixed with 4% formaldehyde and embedded in paraffin. Then histologic analysis of hematoxylin and eosin (H&E)-stained sections was used to examine tumor formation.

### Chromatin immunoprecipitation sequencing (ChIP-seq)

One hundred million cells were fixed with 1% formaldehyde and sonicated for 8 min (10 s on and 15 s off) on ice with a 2-mm microtip with a 40% output control and 90% duty cycle setting. To perform ChIP, sonicated chromatin fragments (150 μl) were diluted 10-fold, and Protein G agarose (60 μl) (Millipore, USA) was added and shaken at 4°C for 2 h. Then, the mixture was briefly centrifuged at 1000 rpm for 5 min at 4°C, and then, the supernatant was collected into a new tube. Anti-H3K18la (PTM-1406) was added to the supernatant and incubated overnight at 4°C. Protein A and protein G magnetic beads (60 μl) (Millipore, USA) were retained in the supernatant for 6 h to pull down the protein at 4°C. The DNA was released from the bound chromatin after cross-linking reversal and proteinase K treatment, precipitated, and diluted in 100 μl of 0.2 M glycine. Purified DNA fragments were constructed and added to ChIP-seq libraries, amplified, and sequenced on an HiSeq 2500 platform (Illumina). The primers used for the quantitative real-time PCR analysis in this study are listed in Supplementary Table S1.

### Cleavage under targets and tagmentation (CUT&Tag) and data analysis

The CUT&Tag assay was performed as described previously with modifications (23). In brief, 1 × 10^5^ cells were washed gently with wash buffer (20 mM HEPES pH 7.5; 150 mM NaCl; 0.5 mM spermidine and 1× protease inhibitor cocktail) two times. Next, 10 μl concanavalin A-coated magnetic beads (Bangs Laboratories) was added to each sample and incubated at RT for 10 min. The unbound supernatant and resuspended cells were removed with Dig Wash Buffer (20 mM HEPES, pH 7.5; 150 mM NaCl; 0.5 mM spermidine; 1× protease inhibitor cocktail; 0.05% digitonin, and 2mM EDTA), and the cells were incubated with a 1:50 dilution of an anti-H3K18la (1406RM, PTM, China) primary antibody or IgG control antibody (12–370, Millipore, USA) overnight at 4 °C. The cells were then incubated first with a 1:100 dilution of a secondary antibody (AP132, Millipore, USA) at RT for 60 min, subsequently with a 1:100 dilution of pA-Tn5 adapter complex at RT for 60 min and with tagmentation buffer (10 mM MgCl_2_ in Dig-med Buffer) at 37°C for 60 min. DNA was purified using phenol–chloroform–isoamyl alcohol extraction and ethanol precipitation. For library amplification, 21 μl of DNA was mixed with 2 μl of a universal i5 primer and a uniquely barcoded i7 primer. A 25-μl volume of NEBNext HiFi 2 × PCR Master Mix was added, and the sample was placed in a thermocycler with a heated lid and processed with the following thermal cycling conditions: 72°C for 5 min (gap filling); 98°C for 30 s; 14 cycles of 98°C for 10 s and 63°C for 30 s; 72°C for 1 min (final extension); and holding at 8°C. Sequencing was performed on an Illumina NovaSeq 6000 system using 150-bp paired-end sequencing following the manufacturer's instructions. The criteria of fold change >2.0 (>1 in the log_2_ ratio value) and *P* value <0.05 were employed to identify differentially accessible peaks.

### MeRIP-seq and data analysis

MeRIP-Seq, referred to here as m^1^A-MeRIP-seq, was performed by Cloudseq Biotech Inc. (Shanghai, China) as per published protocols with minor modifications (16). In brief, RNA was randomly fragmented to approximately 200 nt through RNA Fragmentation Reagents and protein A/G beads were connected to an m^1^A antibody (202003, Synaptic Systems, Germany) by rotating at RT for 1 h. The RNA fragments were incubated with the beads at 4°C for 4 h. Then, the captured RNA was eluted from the beads and extracted with TRIzol Reagent (Invitrogen, USA). Both the input sample and the m^1^A IP sample were subjected to library generation with the NEBNext Ultra RNA Library Prep Kit (New England Biolab, UK). Libraries were qualified by an Agilent 2100 bioanalyzer (Agilent, USA) and sequenced on a NovaSeq 6000 platform (Illumina, USA). The harvested paired-end reads were subjected to quality control using Q30 and 3′ adaptor trimming by cutadapt software (v1.9.3) to remove low-quality reads (24). Clean reads of all libraries were aligned to the reference genome (HG19) using HISAT2 software (v2.0.4) (25). Methylated sites on RNAs (peaks) were identified through MACS software and differentially methylated sites were identified by diffReps (26,27). The peaks identified overlapping with exons of mRNA were chosen. In addition, GO and pathway enrichment analyses were performed using the differentially methylated protein coding genes.

### RNA sequencing (RNA-seq) and data analysis

Total RNA was extracted from the cultured cells by TRIzol Reagent (Invitrogen, USA). A 2100 Bioanalyzer (Agilent Technologies, USA) was used to confirm the integrity of the RNA and a Qubit 2.0 fluorometer with a Qubit RNA Assay Kit (Life Technologies, USA) was employed to measure the concentration. Then, the Illumina TruSeq RNA Sample Prep Kit (San Diego, USA) was used to prepare sequencing libraries and an Illumina HiSeq 2500 platform (San Diego, USA) was used for sequencing. The mRNA levels of the unigenes were identified by TopHat v2.0.9 and Cufflinks and then normalized to FPKM. The criteria of FDR < 0.01 and fold change <0.5 or >2.0 (<−1 or >1 in the log_2_ ratio value, *P* value < 0.05) were used to identify differentially expressed genes.

### Isobaric tags for relative and absolute quantitation (iTRAQ) proteomic analysis

iTRAQ quantitative proteomic analysis was performed by Applied Protein Technology (Shanghai, China) as previously described (28). In brief, wild-type and ALKBH3-deficient cells were lysed with SDT buffer (4% SDS, 100 mM Tris–HCl, 1 mM DTT, pH 7.6). Protein supernatants were subjected to protein digestion, tandem mass tag (TMT) labeling, fractionation, LC–MS/MS analysis, protein identification, and protein quantitation.

### RNA-binding protein immunoprecipitation (RIP)-qPCR

The Magana RIP Quad kit (17-704, Millipore, USA) was employed to examine RNA-binding proteins on specific genes according to the manufacturer's protocols. Briefly, a total of 1.0 × 10^7^ cells were lysed to obtain 200 μl RIP lysate supernatant, of which 15 μl was kept as input control and 150 μl was enriched with antibody- or rabbit IgG-conjugated Protein A/G Magnetic Beads in IP buffer supplemented with RNase inhibitor at 4°C overnight. The immunoprecipitated RNA was digested and purified with the beads. The retrieved RNA and the input control were further analyzed through qPCR. The following primary antibodies were used in the study: anti-YTHDF1 (57530, CST, USA), anti-YTHDF2 (71283, CST, USA) and anti-YTHDF3 (24206, CST, USA).

### Luciferase reporter assay

The DNA fragments of the SP100A-5′UTR containing the wild-type m^1^A motifs and mutant motifs (potential m^1^A was replaced by T) were synthesized and inserted upstream of firefly luciferase of the pmirGLO vector. Cultured 92.1 cells were transfected with pmirGLO-SP100A-WT-luc or pmirGLO-SP100A-MUT-luc. Relative luciferase activity was evaluated 48 h after transfection by the Dual-Luciferase Reporter Assay System (Promega, USA). The sequences are listed in Supplementary Table S2.

### Polysome profiling

Polysome profiling was performed with optimization following the reported protocol (https://www.jove.com/video/51455). In brief, 1.5 × 10^8^ cells were cultured with medium containing 0.1 mg/ml cyclohexamide (CHX) (Sigma-Aldrich, USA) for 5 min at 4°C to freeze the translating ribosomes. Then, the cells were harvested, rinsed with 0.1 mg/ml CHX in precooled PBS and frozen in liquid nitrogen. Next, the cells were homogenized by a liquid nitrogen grinder, lysed with lysis buffer (10 mM Tris–HCl pH 7.5, 100 mM NaCl, 30 mM MgCl_2_, 0.1 mg/ml CHX, 40 U/ml RNase Inhibitor and 1:100 protease inhibitor) and centrifuged. The supernatant was gently added to a linear 10% to 40% w/v sucrose gradient, ultracentrifuged through a Rotor SW41Ti (Beckman, USA), fractioned by a Gradient Station (BioCamp, USA) equipped with an ECONOUV monitor (Bio-Rad, USA) and collected with a Gilson FC203B fraction collector (Mandel Scientific, Canada). RNA was purified from fractions 2 to 17 and subjected to qPCR analysis.

### The Cancer Genome Atlas (TCGA) dataset

To validate the prognostic significance of ALKBH3 and SP100A and determine the correlations between LDHA and ALKBH3, LDHB and ALKBH3, and ALKBH3 and SP100A levels in melanoma, we queried the GEPIA (gepia.cancerpku.cn) and R2 (http://hgserver1.amc.nl/cgibin/r2/main.cgi) to acquire the transcriptional landscape and follow-up information.

### Statistics

Prism 8.0 software (GraphPad, USA) was used to perform statistical analysis. Quantification data are presented as the mean ± SD, and the differences between two groups were compared by unpaired Student's *t* test. Kaplan–Meier curves and log-rank tests were performed for survival analysis. A *P* value <0.05 was considered statistically significant and asterisks denote statistical significance (*P < 0.05, **P < 0.01, ***P < 0.001 and ****P < 0.0001).

## Results

### ALKBH3 is specifically increased in ocular melanoma and is associated with an unfavorable outcome

**Figure 1.**
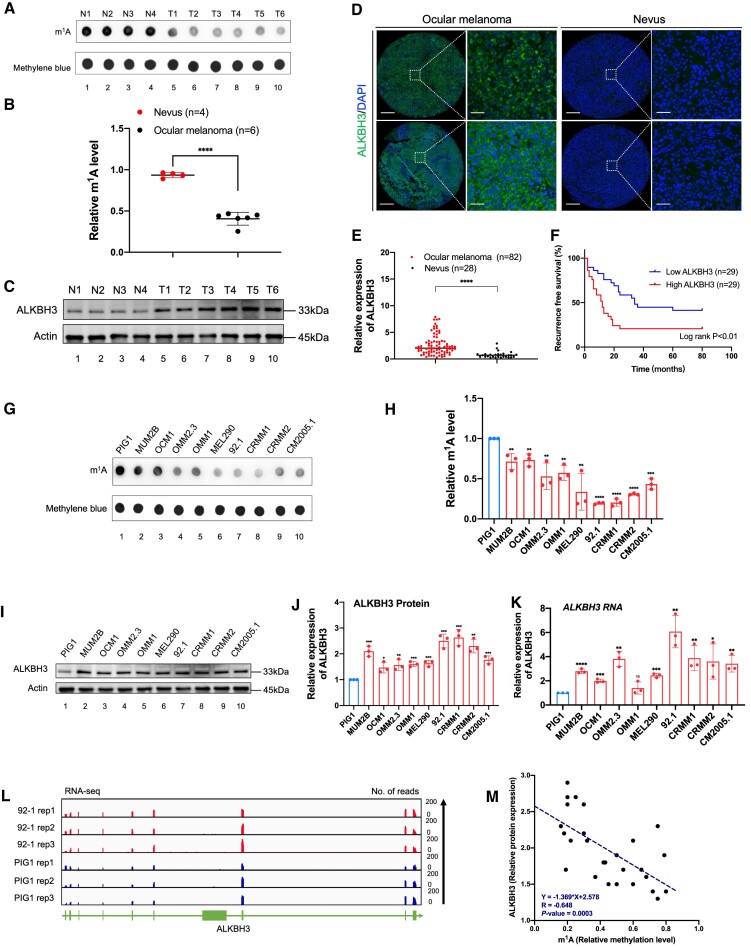
Ocular melanoma exhibits increased ALKBH3 expression and decreased m^1^A levels, which are associated with poor survival. (**A**) Dot blot showing the m^1^A signal relative to the methylene blue signal in tumor and normal samples. The data are representative of experimental triplicates. (**B**) Densitometric analysis showing the expression of m^1^A relative to methylene blue in tumor and normal samples. The data are presented as the mean ± SD of experimental triplicates. Significance was determined by unpaired two-tailed Student's *t* test. ****P < 0.0001. (**C**) Western blot showing ALKBH3 expression relative to ACTB expression in tumor and normal samples. The data are representative of experimental triplicates. (**D**) Immunofluorescence of ALKBH3 (green) and DAPI (blue) in tumor and normal samples. Scale bars: left panel, 100 μm; right panel, 20 μm. (**E**) Statistical results of ALKBH3 levels in normal and tumor tissues. Significance was determined by unpaired two-tailed Student's *t* test. ****P < 0.0001. (**F**) Kaplan–Meier curves of tumor recurrence showing the difference between ocular melanoma patients with low ALKBH3 levels (*n* = 29) and high ALKBH3 levels (*n* = 29). log rank test, *P* < 0.01. (**G**) Dot blot showing the m^1^A signal relative to the methylene blue signal in ocular melanoma cells and normal melanocytes. The data are representative of experimental triplicates. (**H**) Densitometric analysis showing the expression of m^1^A relative to methylene blue in ocular melanoma cells and normal melanocytes. The data are presented as the mean ± SD of experimental triplicates. Significance was determined by unpaired two-tailed Student's *t* test. **P < 0.01, ***P < 0.001, ****P < 0.0001. (**I**) Western blot showing ALKBH3 expression relative to ACTB expression in ocular melanoma cells and normal melanocytes. The data are representative of experimental triplicates. (**J**) Densitometric analysis showing the protein expression of ALKBH3 relative to that of ACTB in ocular melanoma cells and normal melanocytes. The data are presented as the mean ± SD of experimental triplicates. Significance was determined by unpaired two-tailed Student's *t* test. *P < 0.05, **P < 0.01, ***P < 0.001. **(K)** qPCR data showing ALKBH3 expression in ocular melanoma cells relative to PIG1 cells. The data are presented as the mean ± SD of experimental triplicates. Significance was determined by unpaired two-tailed Student's *t* test. *P < 0.05, **P < 0.01, ***P < 0.001, ****P < 0.0001. (**L**) Integrative Genomics Viewer (IGV) tracks for ALKBH3 from RNA-seq data in an ocular melanoma cell line (92.1) and a normal melanocyte cell line (PIG1). Data were obtained from biological triplicates. (**M**) Correlation analysis of the relative protein expression of ALKBH3 and the m^1^A methylation level in ocular melanoma cells and normal melanocytes. Significance was determined by Pearson correlation analysis (*R* = –0.648, *P*= 0.0003).

To determine the role of m^1^A modification in the pathogenesis of ocular melanoma, we first compared the global m^1^A modification levels between ocular melanoma samples (3 CoM samples and 3 UM samples) and 4 control nevus samples. Notably, ocular melanoma samples presented significantly attenuated m^1^A levels, as demonstrated by the anti-m^1^A dot-blot assay (Figure 1A and B). In addition, the demethylase ALKBH3 exhibited increased protein expression in tumors (Figure 1C-E, Supplementary Tables S4 and S5), which is in agreement with the finding of decreased m^1^A levels in ocular melanomas. Moreover, elevated ALKBH3 is associated with unfavorable outcomes (Figure 1F and Supplementary Tables S4 and S6), further underscoring the importance of ALKBH3 in the carcinogenesis of ocular melanoma.

Consistently, in most melanoma cell lines (MUM2B, OCM1, OMM2.3, OMM1, MEL290, 92.1, CRMM1, CRMM2, CM2005.1), a significant decrease in m^1^A levels (Figure 1G and H) and the increase of ALKBH3 levels (Figure 1I–K) were observed when compared with normal pigmented cells (PIG1). Furthermore, high-throughput transcriptome sequencing further confirmed that ALKBH3 is upregulated in ocular melanoma cell lines (Figure 1L). Importantly, among these cells, ALKBH3 presented a significant negative correlation with m^1^A levels, suggesting that upregulated ALKBH3 is responsible for the decreased m^1^A level (Figure 1M).

### ALKBH3 accelerates ocular melanoma oncogenesis *in vitro* and *in vivo*

**Figure 2.**
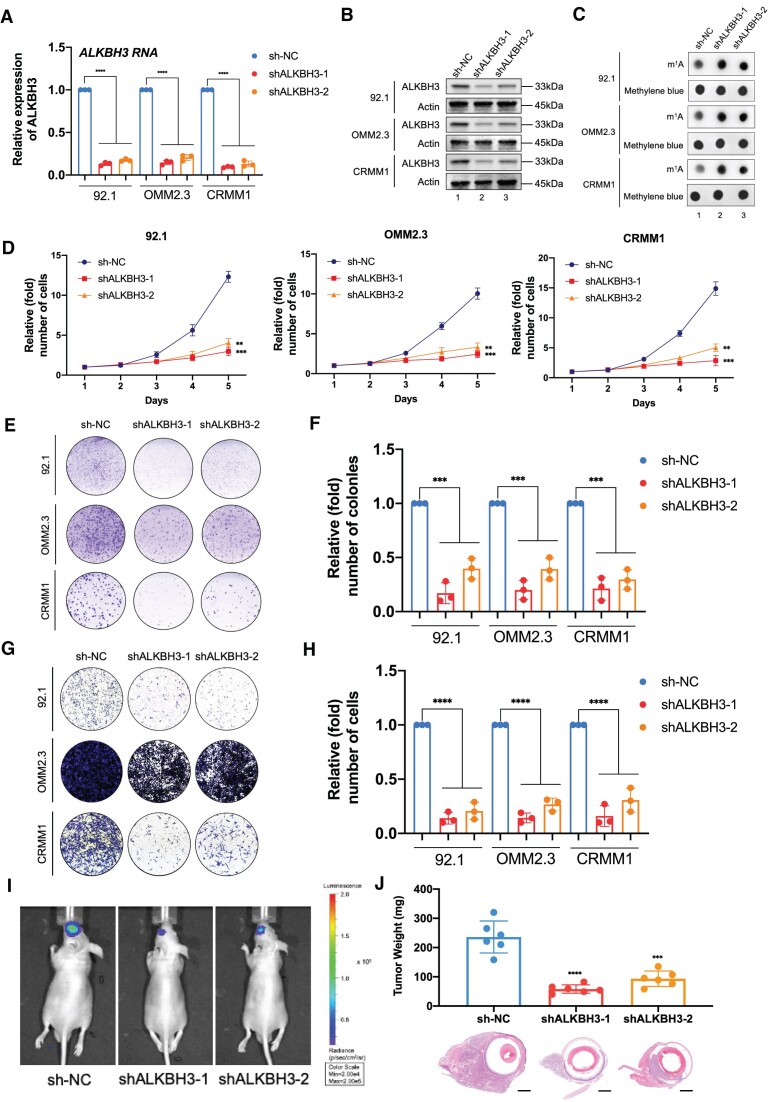
ALKBH3 knockdown increased the m^1^A levels and suppressed ocular melanoma tumorigenesis. (**A**) qPCR data showing ALKBH3 expression in ocular melanoma cells (92.1, OMM2.3 and CRMM1) upon ALKBH3 knockdown. The data are presented as the mean± SD of experimental triplicates. Significance was determined by unpaired two-tailed Student's *t* test. ****P < 0.0001. (**B**) Western blot showing ALKBH3 expression relative to ACTB expression in ocular melanoma cells (92.1, OMM2.3 and CRMM1) upon ALKBH3 knockdown. (**C**) Dot blot showing the m^1^A signal relative to the methylene blue signal in ocular melanoma cells (92.1, OMM2.3 and CRMM1) upon ALKBH3 knockdown. (**D**) A CCK-8 assay was employed to evaluate the proliferation of ocular melanoma cells (92.1, OMM2.3 and CRMM1) upon ALKBH3 knockdown. The data are presented as the mean ± SD of experimental triplicates. Significance was determined by unpaired two-tailed Student's *t* test. **P < 0.01, ***P < 0.001. (**E**) A colony formation assay was employed to evaluate the growth of ocular melanoma cells (92.1, OMM2.3 and CRMM1) upon ALKBH3 knockdown. Representative images from three experimental replicates are shown. (**F**) Statistical analysis of the colony formation assay data in ocular melanoma cells (92.1, OMM2.3 and CRMM1) upon ALKBH3 knockdown. The data are presented as the mean ± SD of experimental triplicates. Significance was determined by unpaired two-tailed Student's *t* test. ***P < 0.001. (**G**) A transwell assay was employed to evaluate the migration of ocular melanoma cells (92.1, OMM2.3 and CRMM1) upon ALKBH3 knockdown. Representative images from three experimental replicates are shown. (**H**) Statistical analysis of the transwell assay data in ocular melanoma cells (92.1, OMM2.3 and CRMM1) upon ALKBH3 knockdown. The data are presented as the mean ± SD of experimental triplicates. Significance was determined by unpaired two-tailed Student's *t* test. ****P < 0.0001. (**I**) Images acquired with an *in vivo* small animal imaging system showing the suppression of bioluminescent signals in orthotopic xenografts derived from ALKBH3-deficient 92.1 cells. Representative images from six biological replicates are shown. The remaining images are provided in Supplementary Figure 1B. (**J**) Histograms of the weights of orthotopic xenografts derived from ALKBH3-deficient 92.1 cells. H&E staining was used to visualize tumor tissues. Representative images from six biological replicates are shown. The data are presented as the mean ± SD. Significance was determined by unpaired two-tailed Student's *t* test. ***P < 0.001, ****P< 0.0001. Scale bar: 200 μm.

To explore the oncogenic function of ALKBH3, we silenced ALKBH3 expression using two individual shRNAs (Figure 2A and B). Concordantly, a significant increase of m^1^A levels was observed in ALKBH3-deficient cells (Figure 2C). Moreover, ALKBH3 silencing resulted in significant attenuation of cell growth (Figure 2D) and colony formation capacity (Figure 2E and F) in all tested ocular melanoma cells. In addition, the knockdown of ALKBH3 led to decreased migration capacity, as demonstrated by the Transwell assay (Figure 2G and H). These data support the fact that ALKBH3 serves as a necessary oncogenic factor for malignant proliferation and metastasis of ocular melanoma *in vitro*. To assess their tumor formation ability *in vivo*, we injected control and ALKBH3-silenced 92.1 melanoma cells (luciferase-labeled) into nude mice and monitored tumor growth in the orthotopic xenograft model (Supplementary Figure S1A and B). Bioluminescence imaging showed a weaker signal intensity in ALKBH3-deficient ocular melanoma cells than in control cells (Figure 2I). In addition, an ∼80% decrease was noted in the average weight of the xenografts in the ALKBH3-silenced group (Figure 2J). Taken together, these experiments demonstrated that ALKBH3 plays an oncogenic role in the tumorigenesis of ocular melanoma *in vitro* and *in vivo*.

### Histone lactylation potentiates the excessive expression of ALKBH3

**Figure 3.**
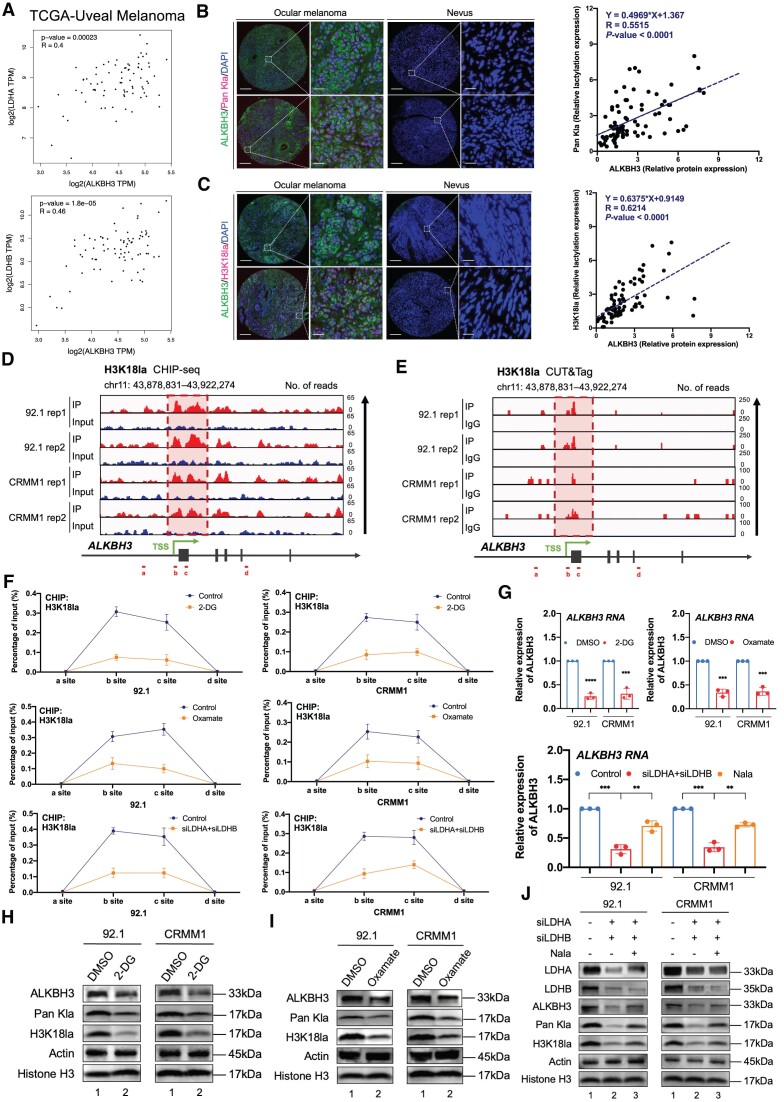
Histone lactylation potentiates the expression of ALKBH3. (**A**) Correlation analysis of ALKBH3 expression and LDHA or LDHB expression in the TCGA-UM cohort (*n* = 80). Significance was determined by *Pearson* correlation analysis (LDHA: R = 0.4, *P* = 0.00023, LDHB: *R* = 0.46, *P* = 0.000018). (**B**) Immunofluorescence of ALKBH3 (green), Pan Kla (red) and DAPI (blue) in tumor and normal samples. Scale bars: left panel, 100 μm; right panel, 20 μm. Correlation analysis of the relative protein expression of ALKBH3 and Pan Kla level in ocular melanoma cells and normal melanocytes. Significance was determined by Pearson correlation analysis (*R*= 0.5515, *P* < 0.0001). (**C**) Immunofluorescence of ALKBH3 (green), H3K18la (red) and DAPI (blue) in tumor and normal samples. Scale bars: left panel, 100 μm; right panel, 20 μm. Correlation analysis of the relative protein expression of ALKBH3 and H3K18la level in ocular melanoma cells and normal melanocytes. Significance was determined by Pearson correlation analysis (*R*= 0.6214, *P* < 0.0001). (**D**) IGV tracks from ChIP-seq analysis showing H3K18la enrichment at the promoter of *ALKBH3*. Sites a–d are distributed in the *ALKBH3* genomic region, and sites b and c are the H3K18la peaks. Biological duplicates were analyzed. (**E**) IGV tracks from CUT&Tag analysis showing H3K18la enrichment at the promoter of *ALKBH3*. Sites a–d are distributed in the *ALKBH3* genomic region, and sites b and c are the H3K18la peaks. Biological duplicates were analyzed. (**F**) ChIP-qPCR assay of H3K18la status in the *ALKBH3*genomic region in ocular melanoma cells (92.1 and CRMM1) upon treatment with histone lactylation inhibitors (2-DG or oxamate) or LDHA/B inhibition. The data are presented as the mean ± SD of experimental triplicates. (**G**) qPCR data showing ALKBH3 expression in ocular melanoma cells (92.1 and CRMM1) upon treatment with histone lactylation inhibitors (2-DG or oxamate) or LDHA/B inhibition. The data are presented as the mean ± SD of experimental triplicates. Significance was determined by unpaired two-tailed Student's *t* test. **P < 0.01, ***P < 0.001, ****P < 0.0001. (**H** and **I**) Western blot showing ALKBH3 expression relative to ACTB expression and Pan Kla, H3K18la expression relative to Histone H3 expression in ocular melanoma cells (92.1 and CRMM1) upon histone lactylation inhibitors (2-DG or oxamate). (**J**) Western blot showing LDHA, LDHB, ALKBH3 expression relative to ACTB expression and Pan Kla, H3K18la expression relative to Histone H3 expression in ocular melanoma cells (92.1 and CRMM1) upon LDHA/B inhibition. Nala, sodium lactate.

To determine the molecular basis underlying the increased expression of ALKBH3, we have queried TCGA database to screen the genes sharing paralleled expression pattern with ALKBH3. According to GO and KEGG analysis, we found ALKBH3-related genes are enriched in several metabolic terms, including oxidative phosphorylation (*P* = 1.36e-05), cellular metabolic process (*P* = 1.50e-05) and carbohydrate derivative metabolic process (*P* = 4.18e-05), suggesting that elevated ALKBH3 RNA expression could be contributed from the metabolic reprogramming (Supplementary Figure S2A and B). Moreover, the lactate producing enzyme LDHA and LDHB showed significant positive correlation with ALKBH3, which indicates the association between the ALKBH3 and lactate (Figure 3A and B, Supplementary Figure S2C and D). Since previous studies have indicated histone lactylation contributes to the activation of oncogenes (29) and ocular melanoma harbor an increased lactylation level (21), we assume that the increase of ALKBH3 levels could be related to the histone lactylation.

We then verified the expression pattern between pan-lactylation and histone lactylation marker (H3K18la) in the ocular melanoma cohorts, which showed a remarkable positive correlation with ALKBH3 protein expression (Figure 3B and C). More importantly, both CUT&Tag and ChIP-seq of H3K18la analysis demonstrated a robust signal of histone lactylation signal, captured in the promoter region of ALKBH3 (deposited in GEO database: GSE242019, Figure 3D and E). Moreover, both histone lactylation inhibitors (oxamate and 2-DG) and LDHA/B inhibition have resulted in a dramatic decreased histone lactylation levels in the promoter region of ALKBH3 (Figure 3F), which subsequently abrogates the RNA (Figure 3G) and protein levels (Figure 3H-J) of ALKBH3 in all tested melanoma cells.

### Multi-omic screening identified SP100A as the downstream candidate of ALKBH3

**Figure 4.**
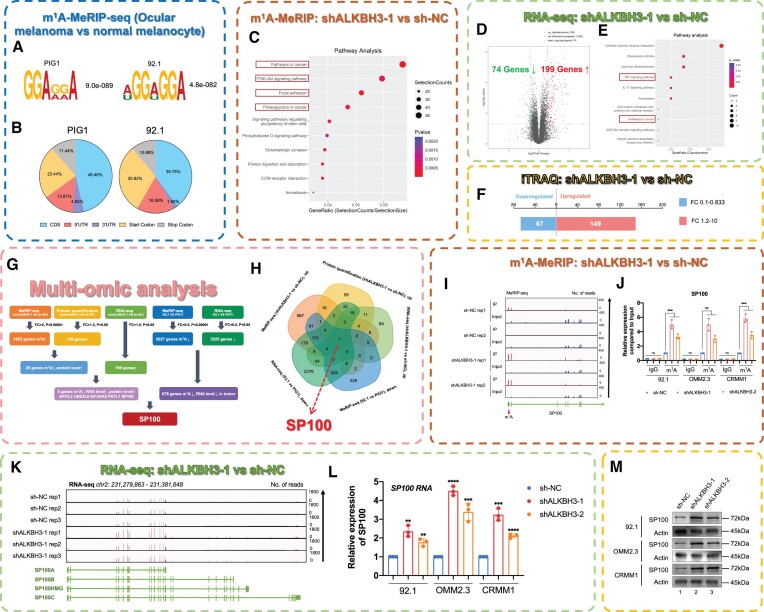
ALKBH3 suppresses SP100 expression by removing its m^1^A modification. (**A**) m^1^A-MeRIP-seq data showing the top enriched motifs within m^1^A peaks identified in ocular melanoma cells and normal melanocytes. (**B**) Pie charts showing the m^1^A peak distribution in different RNA regions (CDS, 5′ UTR, 3′ UTR, start codon and stop codon) in ocular melanoma cells and normal melanocytes. (**C**) KEGG pathway analysis of m^1^A-modified genes in wild-type and ALKBH3-deficient ocular melanoma cells. (**D**) Volcano plots showing ALKBH3-regulated genes in wild-type and ALKBH3-deficient ocular melanoma cells. (**E**) KEGG pathway analysis of ALKBH3-regulated genes in wild-type and ALKBH3-deficient ocular melanoma cells. (**F**) Histogram showing the protein expression of ALKBH3-regulated genes in wide-type and ALKBH3-deficient ocular melanoma cells. (**G** and **H**) Multiomics analysis identified SP100 as a downstream target of ALKBH3. (**I**) IGV tracks from m^1^A-meRIP-seq analysis showing m^1^A enrichment at the 5′UTR of SP100A. Biological duplicates were analyzed. (**J**) m^1^A-MeRIP-qPCR assay of m^1^A status in SP100 in wild-type and ALKBH3-deficient ocular melanoma cells (92.1, OMM2.3 and CRMM1). The data are presented as the mean ± SD of experimental triplicates. Significance was determined by unpaired two-tailed Student's *t* test. **P < 0.01, ***P < 0.001. (**K**) IGV tracks for SP100 from RNA-seq data in wild-type and ALKBH3-deficient ocular melanoma cells. Biological triplicates were analyzed. (**L**) qPCR data showing SP100 RNA expression in ocular melanoma cells (92.1, OMM2.3 and CRMM1) upon ALKBH3 knockdown. The data are presented as the mean ± SD of experimental triplicates. Significance was determined by unpaired two-tailed Student's *t* test. **P < 0.01, ***P < 0.001, ****P < 0.0001. (**M**) Western blot showing SP100 expression relative to ACTB expression in wild-type and ALKBH3-deficient ocular melanoma cells (92.1, OMM2.3 and CRMM1).

We then explored the mechanism underlying the inhibitory effect induced by ALKBH3 silencing in ocular melanoma cells. Since ALKBH3 is responsible for removing m^1^A modifications from RNA, we first performed m^1^A-MeRIP-seq of both ocular melanoma cells and normal melanocytes (deposited in GEO database: GSE213748, Figure 4A). As a result, an average of 16 864 and 10 212 m^1^A peaks were identified from m^1^A-MeRIP-seq libraries generated from normal and tumor cells (Figure 4A and B, blue box). Consistent with the previous m^1^A-meRIP-seq results (30), the m^1^A peaks were enriched in the 5′ UTR, especially near the start codons (Supplementary Figure S3A). Notably, the differentially expressed m^1^A-modified genes were associated with multiple melanoma-related pathways, including DNA replication, mTOR/AMPK signaling and melanin synthesis, suggesting a regulatory role of m^1^A modification in the pathogenesis of ocular melanoma (Supplementary Figure S3B and C).

Additionally, after silencing ALKBH3, we performed a series of comprehensive high-throughput screenings, including m^1^A-MeRIP-seq (deposited in GEO database: GSE213748, Figure 4C, brown box), RNA-seq (deposited in GEO database: GSE213681, Figure 4D and E, Supplementary Figure S4, green box) and proteomic analysis (iTRAQ, Figure 4F) for the 92.1 melanoma cell line. Concordantly, we noticed that the change in m^1^A modification sites was significantly enriched in tumor-related pathways, including PI3K-Akt signaling, focal adhesion and proteoglycan synthesis (Figure 4C). Moreover, the silencing of ALKBH3 results in a remarkable change of the gene expression levels, with 199 upregulated genes and 74 downregulated genes (Figure 4D and E, deposited in GEO database: GSE213681). Consistently, a dramatic genome-wide change in the proteomic levels was observed (149 upregulated and 67 downregulated proteins, Supplementary Table S3, Figure 4F), further underscoring the importance of ALKBH3 in the pathogenesis of ocular melanoma.

Interestingly, combining these multiomics data, we noticed that nuclear autoantigen speckled protein 100 (SP100) was upregulated at both the mRNA and protein levels after silencing ALKBH3 in ocular melanoma cells, following a dramatic change in the m^1^A modification level (Figure 4G-M). Notably, SP100 is responsible for the formation of PML nuclear bodies, predominantly serving as a suppressor of tumorigenesis across various cancer types, encompassing melanoma, glioblastoma, leiomyosarcoma, breast cancer, and laryngeal cancer (31–35). This observation aligns with the findings regarding the inhibitory efficacy observed in ALKBH3-deficient cells. Importantly, SP100 showed decreased m^1^A methylation in ocular melanoma cells compared with normal melanocytes (Supplementary Figure S3D). Interestingly, the decrease in m^1^A modification levels was restored after ALKBH3 inhibition in ocular melanoma cells, as demonstrated by both m^1^A-MeRIP-seq (deposited in GEO database: GSE213748, Figure 4I) and m^1^A-MeRIP-qPCR (Figure 4J). Since recent studies have observed that m^1^A modification of mRNA enhances gene expression and translational efficacy, it is possible that ALKBH3-mediated m^1^A modification is essential for the expression of the tumor suppressor SP100. We then validated that SP100 was significantly upregulated at the mRNA level after inhibiting ALKBH3, as demonstrated by both RNA-seq (deposited in GEO database: GSE213681, Figure 4K and Supplementary Figure S4) and qPCR (Figure 4L). Consistently, SP100 also presented increased protein levels in ALKBH3-inhibited cells, as shown by iTRAQ (Supplementary Figure S5A and B) and Western blot assays (Figure 4M). Consistently, ALKBH3 exhibited a significant negative correlation with SP100 in a group of 87 metastatic melanoma samples (deposited in GEO database: GSE7553, *R* = –0.227, *P*= 0.035, Supplementary Figure S6). Taken together, these data suggest that ALKBH3 may inhibit SP100 expression levels by removing its m^1^A modification.

### SP100A serves as a tumor suppressor in ocular melanoma

**Figure 5.**
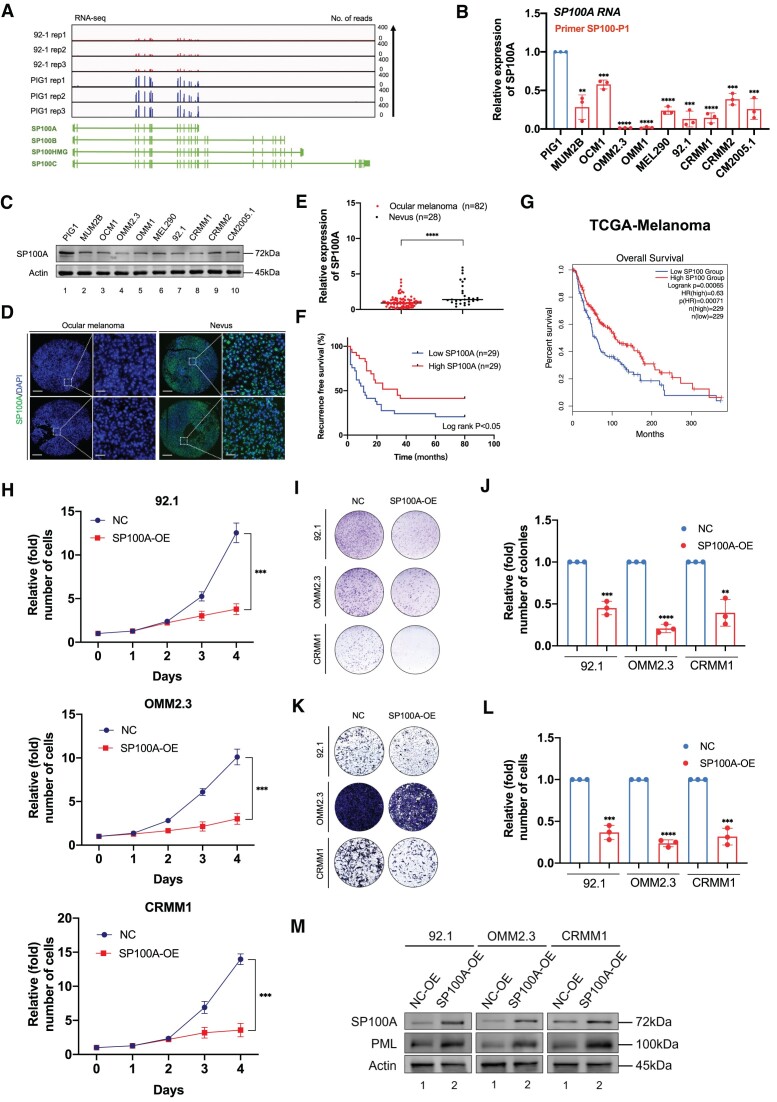
SP100A functions as a tumor suppressor in ocular melanoma. (**A**) IGV tracks for SP100 from RNA-seq data in an ocular melanoma cell line (92.1) and a normal melanocyte cell line (PIG1). Data were obtained from biological triplicates. (**B**) qPCR data showing the SP100A expression in ocular melanoma cells relative to PIG1 cells. The data are presented as the mean ± SD of experimental triplicates. Significance was determined by unpaired two-tailed Student's *t* test. **P < 0.01, ***P < 0.001, ****P < 0.0001. (**C**) Western blot showing SP100A expression relative to ACTB expression in ocular melanoma cells and normal melanocytes. The data are representative of experimental triplicates. (**D**) Immunofluorescence of SP100A (green) and DAPI (blue) in tumor and normal samples. Scale bars: left panel, 100 μm; right panel, 20 μm. (**E**) Statistical results of SP100A levels in normal and tumor tissues. Significance was determined by unpaired two-tailed Student's *t* test. ****P < 0.0001. (**F**) Kaplan–Meier curves of tumor recurrence showing the difference between ocular melanoma patients with low SP100A levels (*n* = 29) and high SP100A levels (*n* = 29). log rank test, *P* < 0.05. (**G**) Kaplan–Meier analysis of the correlations between SP100 expression and overall survival in TCGA melanoma patients stratified by the SP100 expression level: high (top 50th percentile, *n* = 229) and low (bottom 50th percentile, *n* = 229). Significance was determined by a two-sided log-rank test. (**H**) A CCK8 assay was employed to evaluate the proliferation of ocular melanoma cells (92.1, OMM2.3 and CRMM1) upon SP100A overexpression. The data are presented as the mean ± SD of experimental triplicates. Significance was determined by unpaired two-tailed Student's *t* test. ***P < 0.001. (**I**) A colony formation assay was employed to evaluate the growth of ocular melanoma cells (92.1, OMM2.3 and CRMM1) upon SP100A overexpression. Representative images from three experimental replicates are shown. (**J**) Statistical analysis of the colony formation assay data in ocular melanoma cells (92.1, OMM2.3 and CRMM1) upon SP100A overexpression. The data are presented as the mean ± SD of experimental triplicates. Significance was determined by unpaired two-tailed Student's *t* test. **P < 0.01, ***P < 0.001, ****P < 0.0001. (**K**) A transwell assay was employed to evaluate the migration of ocular melanoma cells (92.1, OMM2.3 and CRMM1) upon SP100A overexpression. Representative images from three experimental replicates are shown. (**L**) Statistical analysis of the transwell assay data in ocular melanoma cells (92.1, OMM2.3 and CRMM1) upon SP100A overexpression. The data are presented as the mean ± SD of experimental triplicates. Significance was determined by unpaired two-tailed Student's *t* test. ***P < 0.001, ****P < 0.0001. (**M**) Western blot showing PML expression relative to ACTB expression in wild-type and SP100A-overexpressing ocular melanoma cells (92.1, OMM2.3 and CRMM1).

Significantly, in the context of viral infection response, four prominent isoforms of SP100 (namely SP100A, SP100B, SP100C, and SP100HMG) have been discerned (36), prompting us to undertake a comparative analysis of the expression levels exhibited by these transcripts. It is worth mentioning that SP100A exhibits a commonality with SP100B, SP100C and SP100HMG in terms of the initial 15 exons (1–1562 bp), while a disparity of 395-bp in length is observed in the final exon (Supplementary Figure S7A). The RNA-seq data obtained from ocular melanoma cells (92.1) and normal melanocytes (PIG1) revealed a prominent signal in SP100A, while the signal in the exons not included in SP100A was negligible (Figure 5A). Furthermore, we proceeded to conduct qPCR using various primers. These primers encompassed a set designed specifically for SP100A (referred to as SP100-P1), and another set that detects SP100B, SP100C, and SP100HMG (referred to as SP100-P2). The RNA expression of SP100A was observed in ocular melanoma cells and normal pigmented cells (Figure 5B). Since human breast cancer cell line ZR-75-1 was reported to express SP100HMG, we employed ZR-75–1 cells as the positive control (37). Importantly, SP100B/SP100C/SP100HMG could be only detected in ZR-75-1, while undetected in other cell lines (Supplementary Figure S7B). Moreover, the four isoforms have discrepancies in the molecular weight (Supplementary Figure S8A). The full western blots revealed the specific protein expression of SP100A (∼72 kDa) in all tested cell lines (Supplementary Figure S8B and C), which is consistent with the molecular weight of SP100A in the previous studies (18). The findings suggest that the SP100A is abundantly expressed in our experimental context, whereas the expression of other isoforms (SP100B, SP100C and SP100HMG) is negligible.

Notably, a significantly decreased RNA expression level of SP100A was also observed in ocular melanoma cells when compared to the normal pigmented cells, as demonstrated by RNA-seq (Figure 5A), qPCR (Figure 5B) and Western blot assays (Figure 5C). To fully uncover the function of SP100A in ocular melanoma, we then determined SP100A expression in clinical samples of ocular melanoma. Notably, we found that SP100A was significantly decreased in the ocular melanoma samples (Figure 5D and E, Supplementary Tables S4 and S5). More importantly, the loss of SP100A was associated with an unfavorable outcome in both our cohort (Figure 5F and Supplementary Tables S4 and S7) and the TCGA cohort (Figure 5G).

Moreover, according to the single-cell analysis of ocular melanoma samples (Supplementary Figure S9A, GSE139829, using CancerSEA platform (38)), SP100 expression is related to a decreased score of cancer-activation hallmarks, including invasion (*R* = –0.33, *P*< 0.001), metastasis (*R* = –0.28, *P*< 0.001), cell cycle activation (*R* = –0.19, *P*< 0.001), proliferation (*R* = –0.16, *P*< 0.001) and epithelial-to-mesenchymal transition (*R* = –0.16, *P*< 0.001). Collectively, these data indicate that SP100A is downregulated and potentially inhibits several oncogenic events in ocular melanoma.

Since SP100A was downregulated in ocular melanoma, we exogenously overexpressed SP100A in three ocular melanoma cell lines (Supplementary Figure S9B). Interestingly, all tested ocular melanoma cells presented with attenuated proliferation ability after overexpressing SP100A (Figure 5H). Moreover, SP100A-overexpressing melanoma cells formed smaller and fewer colonies than the control group (Figure 5I and J). In addition, a significant inhibition of cancer metastasis capacity was observed after introducing SP100A in ocular melanoma cells (Figure 5K and L).

Most importantly, since SP100A serves as the molecular scaffold within PML bodies (39), we then assessed the PML expression in SP100A-overexpressing cells. As a result, the exogenous overexpression of SP100A resulted in a noteworthy increase in the protein level of PML (Figure 5M). Concordantly, the IF analysis demonstrated a notable augmentation in the presence of PML bodies in SP100A-overexpressing cells (Supplementary Figure S10A). Collectively, this data further confirmed that SP100A is essential for PML expression, consistent with previous research (40). Moreover, ALKBH3-deficient cells exhibited a substantial elevation in PML bodies (Supplementary Figure S10B), which is in alignment with the pivotal role of ALKBH3 in the regulation of SP100A expression. Taken together, these gain-of-function data revealed that SP100A is responsible for the formation of PML nuclear condensates, serving as a tumor suppressor in ocular melanoma.

### Silencing of SP100A partially compromised tumor inhibition efficacy in ALKBH3-deficient cells

**Figure 6.**
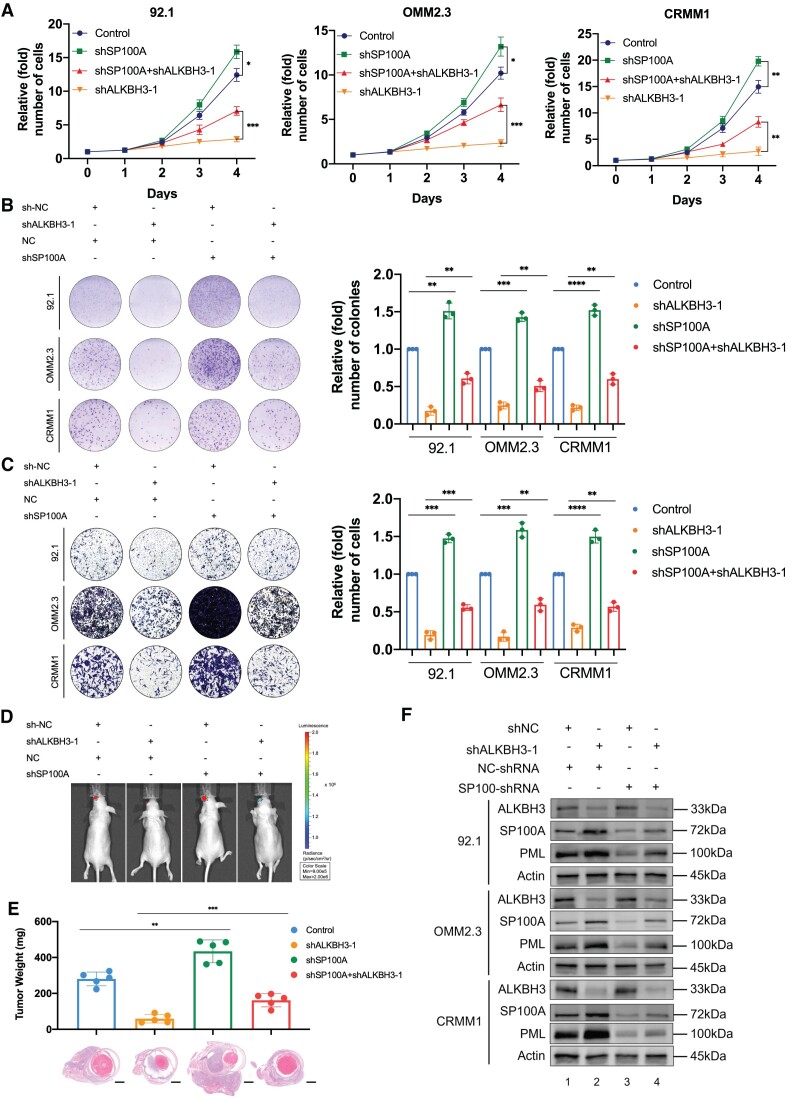
The anticancer effects of ALKBH3 knockdown were partially blocked by SP100A silencing. (**A**) A CCK8 assay was performed to assess the proliferation of ALKBH3-deficient ocular melanoma cells (92.1, OMM2.3 and CRMM1) upon SP100A silencing. The data are presented as the mean ± SD of experimental triplicates. Significance was determined by unpaired two-tailed Student's *t* test. *P < 0.05, **P < 0.01, ***P < 0.001. (**B**) A colony formation assay was performed to assess the growth of ALKBH3-deficient ocular melanoma cells (92.1, OMM2.3 and CRMM1) upon SP100A silencing. Representative images from three experimental replicates are shown. The data are presented as the mean ± SD. Significance was determined by unpaired two-tailed Student's *t* test. **P < 0.01, ***P < 0.001, ****P < 0.0001. (**C**) A transwell assay was performed to assess the migration of ALKBH3-deficient ocular melanoma cells (92.1, OMM2.3 and CRMM1) upon SP100A silencing. Representative images from three experimental replicates are shown. The data are presented as the mean ± SD. Significance was determined by unpaired two-tailed Student's *t* test. **P < 0.01, ***P < 0.001, ****P < 0.0001. (**D**) Images acquired with an *in vivo* small animal imaging system showing the suppression of bioluminescent signals in orthotopic xenografts derived from ALKBH3-deficient 92.1 cells upon SP100A silencing. Representative images from five biological replicates are shown. The remaining images are provided in Supplementary Figure S12B. (**E**) Histograms of the weights of orthotopic xenografts derived from ALKBH3-deficient 92.1 cells upon SP100A silencing. H&E staining was used to visualize tumor tissues. Representative images from five biological replicates are shown. The data are presented as the mean ± SD values. Significance was determined by unpaired two-tailed Student's *t* test. **P < 0.01, ***P < 0.001. Scale bar: 200 μm. (**F**) Western blot showing ALKBH3, SP100A, PML expression relative to ACTB expression in ocular melanoma cells (92.1, OMM2.3 and CRMM1) upon ALKBH3 and SP100A knockdown.

To further verify the relationship between ALKBH3 and SP100A expression, we further altered SP100A expression after ALKBH3 inhibition in ocular melanoma cells by transfecting a reported shRNA against SP100A (41). As expected, SP100A expression was significantly decreased after transfecting SP100A-shRNA into three ocular melanoma cells at both the RNA (Supplementary Figure S11A) and protein (Supplementary Figure S11B) levels. In cell growth, the depletion of SP100A partially rescued (∼50–60%) the inhibition guided by ALKBH3 inhibition (Figure 6A, red line), and SP100A-deficient cells were much more resistant to the deprivation of ALKBH3 (Figure 6A). Similarly, SP100A-silenced cells presented with more colonies than the control group (Figure 6B, red and orange column). Moreover, SP100A inhibition significantly enhanced cell migration ability and compromised the inhibitory efficacy in ALKBH3-deficient melanoma cells (Figure 6C). Most importantly, the knockdown of SP100A further rescued orthotopic tumor formation in ALKBH3-depleted melanoma cells (Figure 6D and E, Supplementary Figure S12A and B). Consistently, the stable knockdown of SP100A resulted in a compromised expression of PML expression in both wild-type (Figure 6F, lanes 1 and 3) and ALKBH3-deficient ocular melanoma cells (Figure 6F, lanes 2 and 4). Together, these results suggested that ALKBH3 promotes ocular melanoma through the decrease of SP100A-mediated PML bodies both *in vivo* and *in vitro*.

### m^1^A modification of SP100A enhances its RNA stability and translational efficacy

**Figure 7.**
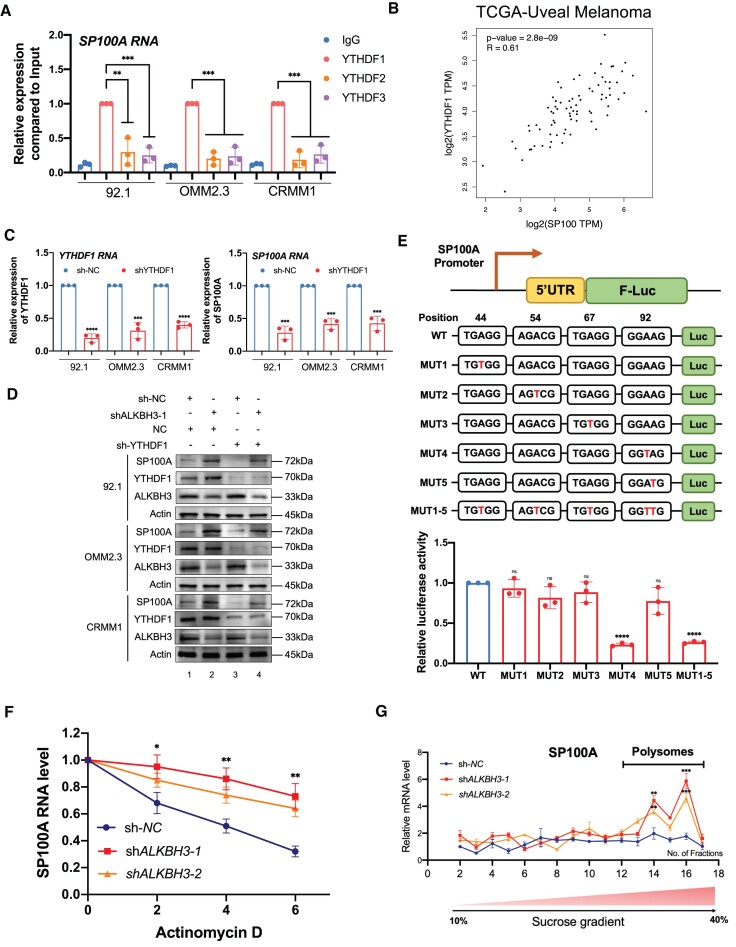
m^1^A modification of SP100A increases its RNA stability and translational efficacy. (**A**) RIP-qPCR assay of SP100A expression in ocular melanoma cells (92.1, OMM2.3 and CRMM1) by YTHDF1, YTHDF2 and YTHDF3. The data are presented as the mean ± SD of experimental triplicates. Significance was determined by unpaired two-tailed Student's *t* test. **P < 0.01, ***P < 0.001. (**B**) Correlation analysis of SP100A expression and YTHDF1 expression in the TCGA-UM cohort (*n* = 80). Significance was determined by *Pearson* correlation analysis (*R* = 0.61, *P* < 0.0001). (**C**) qPCR data showing YTHDF1 RNA expression and SP100A RNA in ocular melanoma cells (92.1, OMM2.3 and CRMM1) upon YTHDF1 knockdown. The data are presented as the mean ± SD of experimental triplicates. Significance was determined by unpaired two-tailed Student's *t* test. ***P < 0.001, ****P< 0.0001. (**D**) Western blot showing YTHDF1, SP100A, ALKBH3 expression relative to ACTB expression in ocular melanoma cells (92.1, OMM2.3 and CRMM1) upon ALKBH3 and YTHDF1 knockdown. (**E**) The luciferase reporter gene assay demonstrated the relative luciferase activity of the wild-type and six mutant SP100A 5′UTR reporter vectors. The data are presented as the means ± SD of experimental triplicates. Significance was determined by unpaired two-tailed Student's *t* test. ****P < 0.0001. (**F**) Half-life of SP100A in wild-type and ALKBH3-deficient 92.1 cells treated with actinomycin (5 g/ml) for 0–6 h. (**G**) Polysome profiling assays of 92.1 cells with or without ALKBH3 knockdown. RNAs in different ribosome fractions were extracted and subjected to qPCR analysis. Data are shown as the mean ± SD. Significance was determined by unpaired two-tailed Student's *t* test. ***P < 0.001, ****P < 0.0001.

We then explored the epigenetic mechanisms underlying the m^1^A modification of SP100A. Since previous studies have demonstrated that YTHDF proteins are responsible for the recognition of m^1^A methylation, we first tested the RNA-binding status of SP100A mRNA with YTHDF1, YTHDF2 and YTHDF3. RNA-immunoprecipitation analysis demonstrated that YTHDF1 specifically recognizes SP100A mRNA; however, YTHDF2 and YTHDF3 only presented with limited interaction intensities (Figure 7A). Moreover, YTHDF1 exhibited a salient positive correlation (*R* = 0.61, *P*< 0.0001) with SP100A expression in the TCGA cohort (Figure 7B), which is in perfect alignment with the hypothesis that YTHDF1 is necessary for the recognition of SP100A. Moreover, YTHDF1 silencing dramatically inhibited SP100A expression and fully rescued ALKBH3 silencing-mediated SP100A levels elevation (Figure 7C and D). Taken together, these data showed that YTHDF1 functions as the reader protein of SP100A.

We then determined the specific m^1^A modification site of SP100A mRNA. In the 5′ UTR of SP100A, we found five potential m^1^A sites according to the identified peak (Figure 4I, Supplementary Figure S3D) [c.44A (TGTGG), c.54A (AGACG) and c.67A (TGAGG), and c. 92A/c. 93A (GGAAG)] and mutated each A into a T. We then cloned the corresponding wild-type and mutated 5′ UTRs into the pmirGLO vector (Figure 7E, upper panel). The luciferase reporter gene assay demonstrated that c.A92T presented a decreased signal, while the signals in the other mutated groups remained unchanged (Figure 7E). Furthermore, we performed RIP-qPCR to identify the interaction frequency between YTHDF1 and reporter transcripts (F-luc). We observed that pmirGLO-MUT4 (c.A92T) transcript presented a decreased binding affinity between YTHDF1 and F-Luc, while other transcripts remained unchanged (Supplementary Figure S13A). This result agrees with the previous observation that YTHDF1 directly interacts with m^1^A methylated sequences, which was regarded as ‘Reader’ for m^1^A in RNA (42,43). Moreover, we found that ALKBH3-deficient cells presented with enhanced RNA stability of SP100A, which agrees with the increase of SP100A RNA expression after ALKBH3 depletion (Figure 7F). Importantly, polysome profiling in 92.1 cells showed that stable knockdown of ALKBH3 resulted in pronounced SP100A mRNA abundance in polysome portions (Figure 7G, Supplementary Figure S13B), which generally harbor effective translation capacity (44). This observation is in alignment with a previous conclusion that m^1^A modification in early exons leads to enhanced translational capacity (30). Notably, the nascent SP100A RNA expression remained unaltered upon ALKBH3 knockdown (Supplementary Figure S13C). Collectively, these results demonstrated that the m^1^A RNA methylation of SP100A mRNA contributes to increased RNA stability and translational capacity post-transcriptionally.

## Discussion

In this study, we described that lactylation-driven ALKBH3 elevation contributes to the SP100A m^1^A demethylation and inhibits its RNA and protein expression. The decreased expression of SP100A further results in the loss of PML bodies, which initiate the progression of ocular melanoma. Notably, PML bodies are pan-cancer pleiotropic tumor suppressors that form phase-separated liquid-like condensates and are organized by multivalent interactions among proteins and RNA molecules. Albeit m^1^A RNA modification plays key roles in the regulation of RNA metabolism and gene expression, the relationship between m^1^A modification and PML formation remains enigmatic. In this study, we revealed for the first time that ALKBH3-mediated m^1^A modification plays an inhibitory role in the formation of the PML body, which bridges our understanding of dynamic RNA modifications and phase-separation events in cancers.

Loss of homeostasis of the m^1^A modification has been associated with several oncogenic hallmarks. For example, ALKBH3-mediated m^1^A demethylation of ATP5D facilitates its expression in metabolic reprogramming during carcinogenesis (9). Moreover, ALKBH3 loss induces m^1^A hypermethylation of the transcriptome in Hodgkin lymphoma, which is associated with poor clinical outcome (14). In addition, m^1^A modification regulates colony-stimulating factor 1 expression in breast and ovarian cancer cells, which indicates that m^1^A modification is responsible for regulating immune surveillance in cancer (45). m^1^A modification also modulates tRNA stability, which plays an important role in regulating apoptosis (15). In this study, we show that the m^1^A modification of SP100A is responsible for the formation of tumor-suppressive PML bodies. To our knowledge, this is the first study to demonstrate that m^1^A is necessary for tumor suppressor gene expression, which expands the current understandings of dynamic m^1^A function.

Histone lactylation, serving as a metabolic stress-related histone modification, promotes cardiac repair, macrophage polarization and facilitates carcinogenesis and progression (29,46). For example, histone lactylation fuels early activation of the reparative transcriptional response in monocytes, regulating immune homeostasis in cardiac repair process (47). Moreover, enhanced histone lactylation also contributes M1 macrophage polarization during bacterial infection (29). In this study, we revealed that histone lactylation is also responsible for the oncogenic activation of the ALKBH3, contributing to the hypo-m^1^A status in cancers. While lactylation inhibitors such as oxamate and 2-DG, along with LDHA/B inhibition, demonstrate effective regulation of lactylation levels, they also elicit additional undesirable consequences (48). Furthermore, due to the shared regulators (eg. EP300 and HDACs) between histone lactylation and other forms of histone modifications (49), the ability to specifically modulate histone lactylation level is constrained.

SP100 was first identified as a nuclear autoimmune antigen and is a constituent of the nuclear body, serving as an important tumor suppressor in diverse cancers, including pancreatic cancer, leukemia and glioma (32,39,50). SP100 plays a vital role in the response to interferon, regulating immune surveillance in carcinogenesis (51,52). Moreover, SP100 interacts with the ETS1 transcription factor, reducing ETS1-DNA binding and impairing ETS1 transcriptional activity on the promoter of several oncogenes (34,53). SP100A, as an important functional isoform of SP100, is a tumor suppressor that activates p53-dependent transcription and counteracts E1A/E1B-55K-mediated transformation (54).

In addition, SP100 was discovered to exert its tumor-suppressive effects when it functions as a constituent of PML nuclear bodies. Notably, PML nuclear bodies have been recognized as tumor suppressors due to their ability to impede the progression of the cell cycle, initiate apoptosis, and inhibit angiogenesis in cancerous cells (55). For example, PML nuclear bodies mediate the p53 response to oncogenic signals and induce senescence. The integrity of the PML nuclear bodies is essential for p53-dependent senescence (56). Moreover, PML nuclear bodies regulate TNFα-induced cell death in cancer cells and suppress capillary tube formation and migration in endothelial cells (57). In this study, we revealed for the first time that SP100A also function as a tumor suppressor in ocular melanoma, providing a novel therapeutic target for vision- and life- threatening ocular melanoma.

Notably, loss of SP100A only partially rescues the ALKBH3-knockdown inhibitory effect, which may be attributed to the diversified functions of ALKBH3 in different RNA categories. ALKBH3, as the demethylase of m^1^A methylation, mediates m^1^A methylation in tRNAs, rRNAs, mRNAs, and mitochondrial RNAs (3). For example, ALKBH3-mediated tRNA demethylation efficiently promotes cancer progression via induction of tRNA-derived small RNAs, which prevents apoptosis induced by cytochrome C (15). Moreover, the m^1^A modification of tRNA also leads to liver tumorigenesis by regulating cholesterol metabolism (8). However, the function of ALKBH3-mediated m^1^A demethylation of tRNAs/rRNAs in the oncogenesis of ocular melanoma remains enigmatic, which warrants further explorations.

In summary, our results reveal a completely novel model of tumorigenesis in which m^1^A modification of SP100A mRNA promotes the formation of PML nuclear condensates. Additionally, this study for the first time reveals that m^1^A is responsible for the activation of tumor suppressor gene and unveils crosstalk between histone lactylation, mRNA m^1^A modification, and phase-separation-mediated condensate formation, thereby providing a novel therapeutic method of targeted m^1^A reprogramming for efficient for tumor treatment.

## Supplementary Material

gkad1193_Supplemental_File

## Data Availability

The raw sequence data reported in this paper, including CUT&Tag, ChIPseq, m^1^A-meRIP-seq and RNA-seq data, have been deposited in the Gene Expression Omnibus database (GSE176345, 213681, 213748 and 242019).
