# Supplementary material for: Histone lactylation-boosted ALKBH3 potentiates tumor progression and diminished promyelocytic leukemia protein nuclear condensates by m1A demethylation of SP100A
Source: Nucleic Acids Res. 2023 Dec 20;52(5):2273–89. doi: 10.1093/nar/gkad1193 (PMC10954454; doi:10.1093/nar/gkad1193)
Supplement: gkad1193_Supplemental_File [file gkad1193_supplemental_file.pdf]

Supplementary material

Histone lactylation-boosted ALKBH3 potentiates tumor progression and diminished promyelocytic leukemia protein nuclear condensates by m^1A demethylation of SP100

Xiang Gu[#], Ai Zhuang[#], Jie Yu[#], Ludi Yang[#], Shengfang Ge, Jing Ruan, Renbing Jia[*], Xianqun Fan[*], Peiwei Chai[*]

Department of Ophthalmology, Shanghai Key Laboratory of Orbital Diseases and Ocular Oncology, Shanghai Ninth People's Hospital, Shanghai Jiao Tong University School of Medicine, Shanghai, 20025, People's Republic of China.

[#]These authors contributed equally to this work.

[*]Corresponding author: renbingjia@sjtu.edu.cn (R.J.); fanxq@sjtu.edu.cn (X.F.); chaipeiwei123@sjtu.edu.cn (P.C.).

Supplementary material files include 15 Figures and 7 Tables

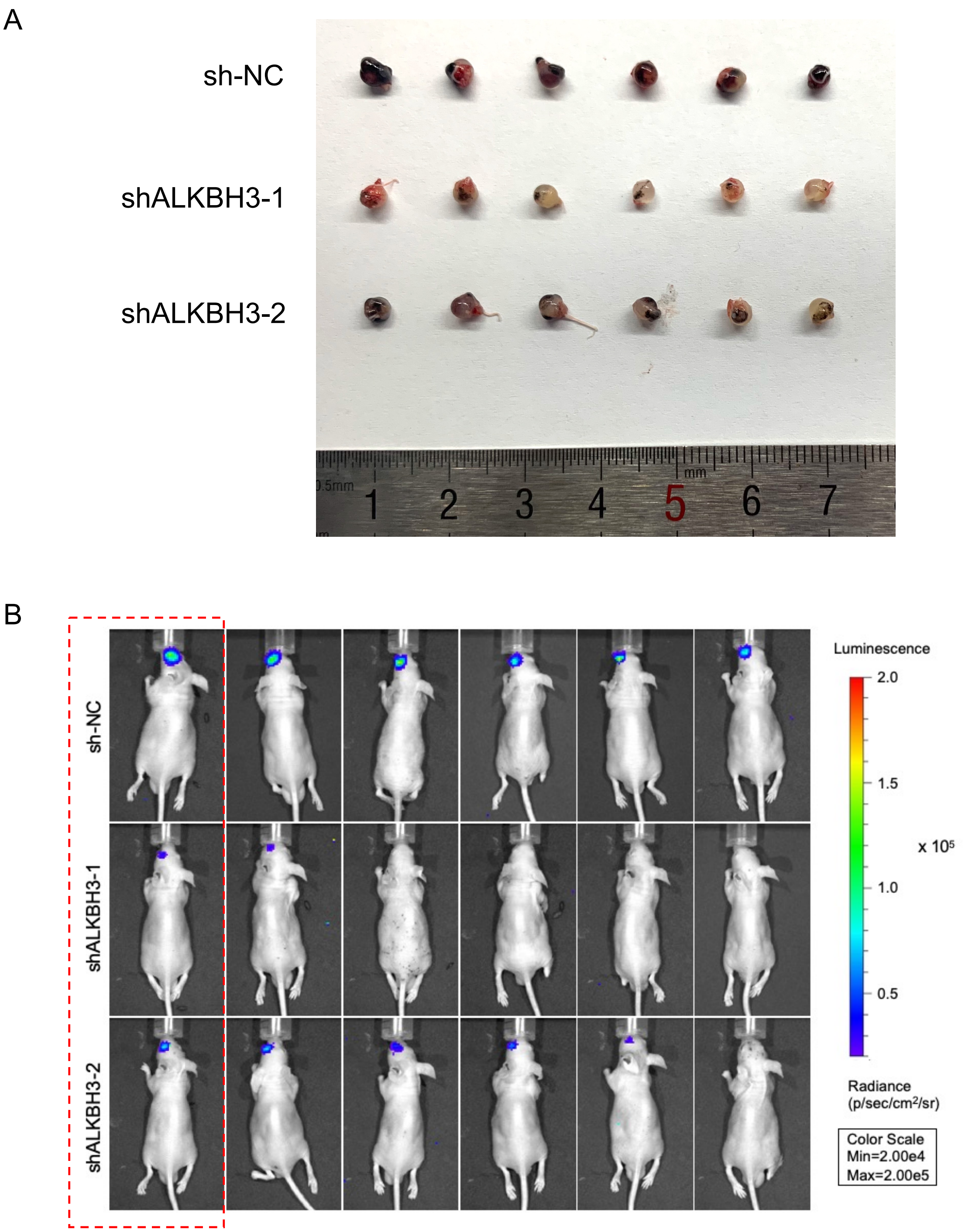

Supplementary Figure S1. ALKBH3 knockdown inhibited ocular melanoma tumorigenesis in vivo. (**A**) Images showing suppression of tumors in orthotopic xenografts derived from ALKBH3-deficient 92.1 cells. The data were obtained from six experimental replicates. (**B**) Images acquired with the small animal imaging system showing suppression of bioluminescent signals in orthotopic xenografts derived from ALKBH3-deficient 92.1 cells. The data were obtained from six experimental replicates.

A

TCGA - Melanoma		
**ALKBH3-related genes**	**Correlated gene ratio**	***P***
Oxidative phosphorylation	23.08%	****
Cellular metabolic process	11.27%	****
Carbohydrate derivative metabolic process	13.89%	****
Glycosaminoglycan metabolic process	18.35%	**
Cellular macromolecule metabolic process	16.11%	**
Small molecule metabolic process	12.38%	**
Primary metabolic process	11.00%	**
Nucleotide metabolic process	13.80%	**
Melanin metabolic process	33.33%	**
ATP metabolic process	15.71%	**
Cellular macromolecule metabolic process	11.00%	*

B

TCGA - Melanoma - Mini ontology analysis				
**Category**	**Cut-off**	**Total**	**%**	***P***
All	2458	20165	12.2	1.000
Cell cycle	45	492	9.1	0.039
Development	261	1605	16.3	6.14e-07
Differentiation	120	682	17.6	1.60e-05
Drug target	170	1151	14.8	7.46e-03
Membrane	656	4919	13.3	0.014

C

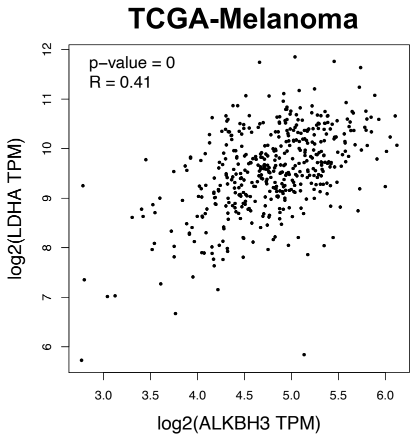

D

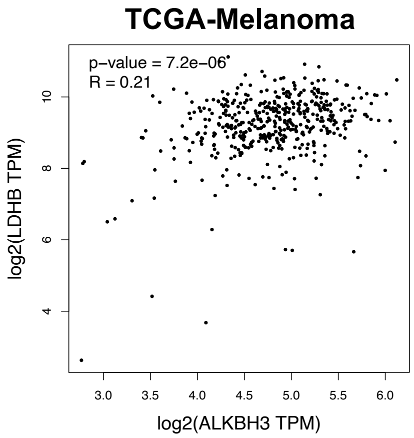

Supplementary Figure S2. ALKBH3-related genes are enriched in metabolic terms. (**A**) GO analysis of ALKBH3-related genes in the TCGA-Melanoma cohort (n=470). (**B**) Mini ontology analysis of ALKBH3-related genes in the TCGA-Melanoma cohort. (**C** and **D**) Correlation analysis of ALKBH3 expression and LDHA (**C**) or LDHB (**D**) expression in the TCGA-Melanoma cohort. Significance was determined by *Pearson* correlation analysis (LDHA: R = 0.41, LDHB: R = 0.21).

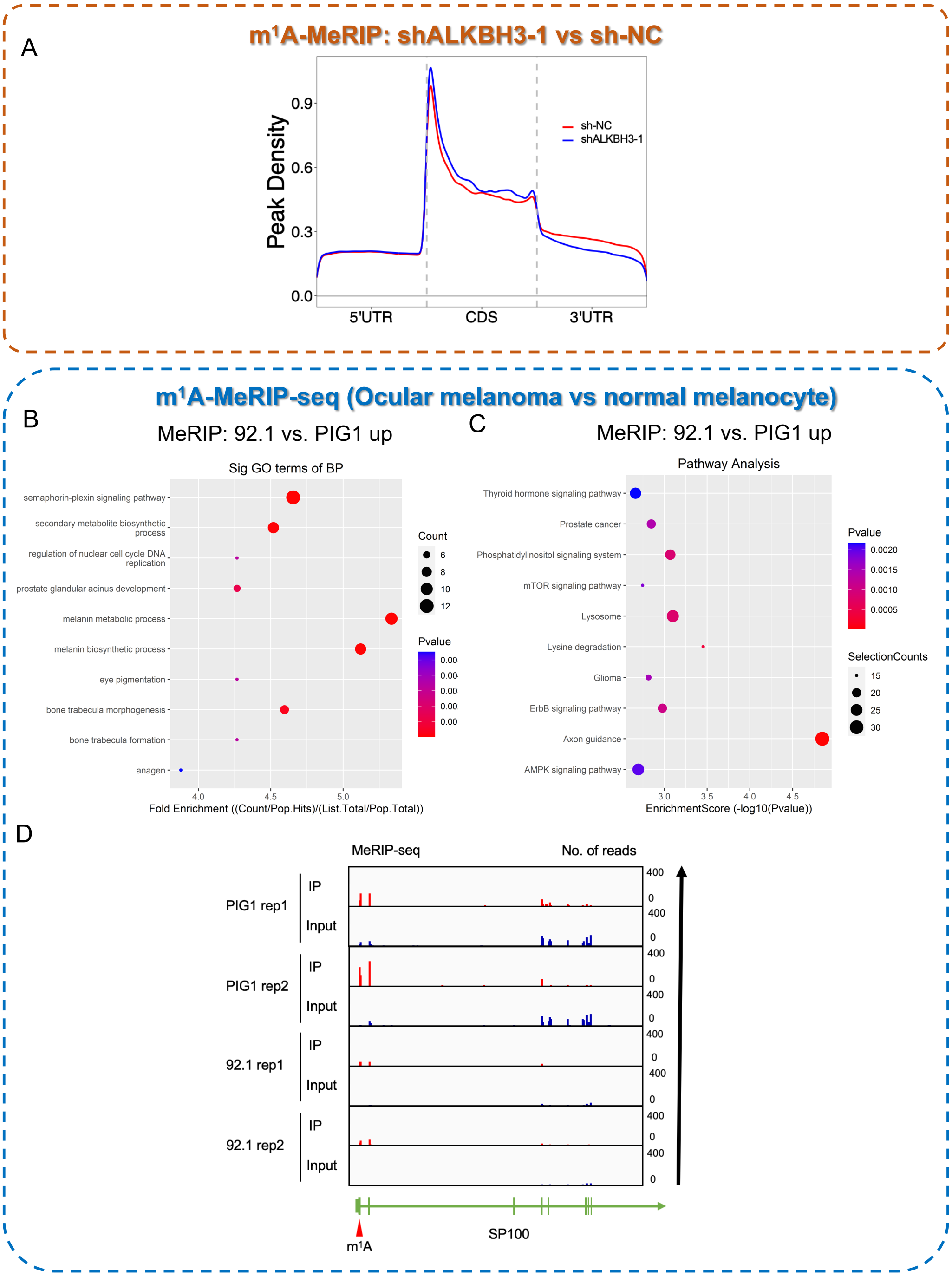

Supplementary Figure S3. m^{1}A-modified genes were associated with tumor-related pathways. (**A**) m^{1}A-meRIP-seq data showing the peak density of m^{1}A sites. Biological duplicates were analyzed. (**B**) GO analysis of m^{1}A-modified genes in ocular melanoma cells and normal melanocytes. (**C**) KEGG pathway analysis of m^{1}A-modified genes in ocular melanoma cells and normal melanocytes. (**D**) IGV tracks from m^{1}A-meRIP-seq analysis showing m^{1}A enrichment at the 5’UTR of SP100. Biological duplicates were analyzed.

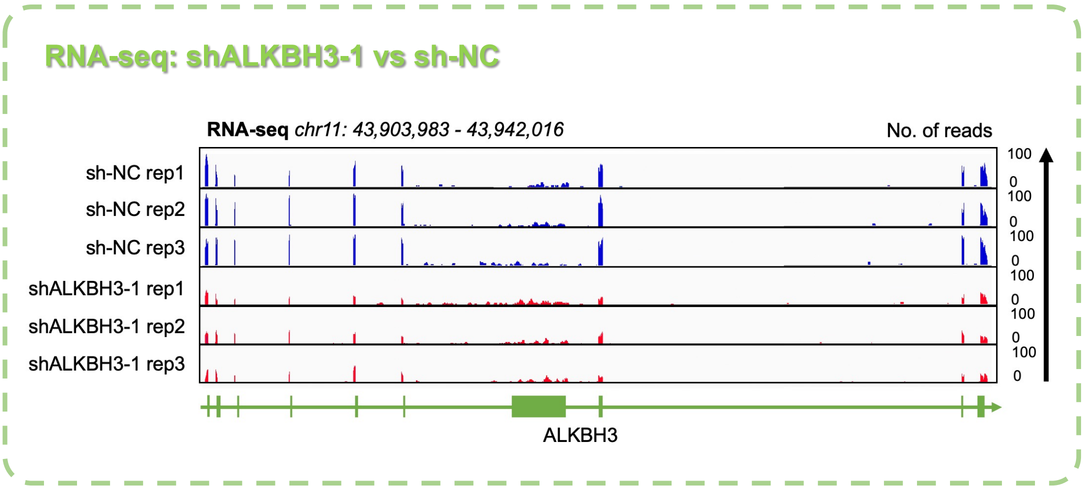

Supplementary Figure S4. The expression of ALKBH3 upon ALKBH3 knockdown. IGV tracks from RNA-seq analysis showing ALKBH3 expression in wild-type and ALKBH3-deficient ocular melanoma cells. Biological triplicates were analyzed.

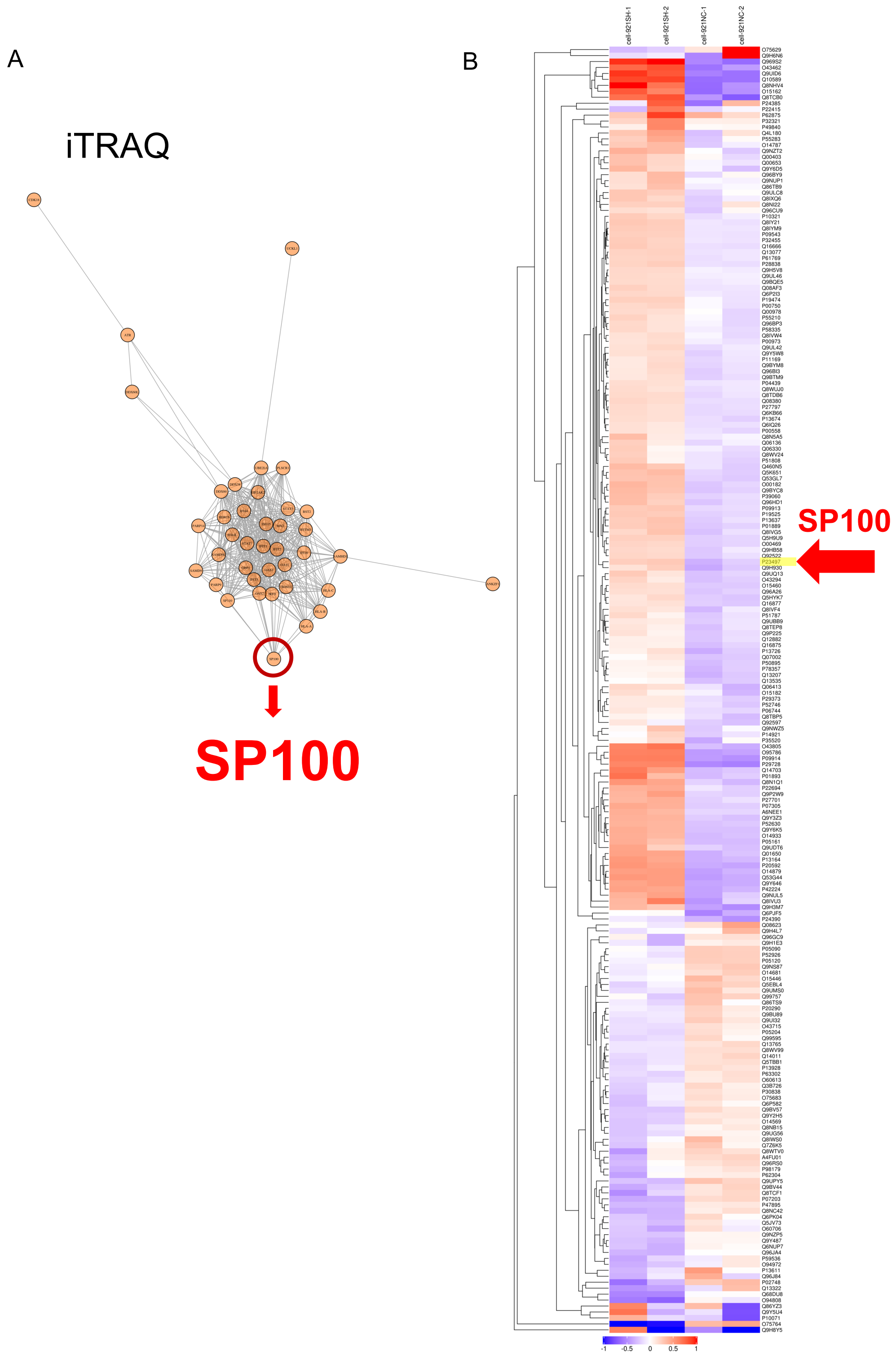

Supplementary Figure S5. SP100 presented with increased protein levels in ALKBH3-deficient cells. (**A**) Protein-protein interaction network analysis showing the protein expression of ALKBH3-regulated genes in wild-type and ALKBH3-deficient ocular melanoma cells. (**B**) Heatmap showing the differentially expressed proteins in wild-type and ALKBH3-deficient ocular melanoma cells.

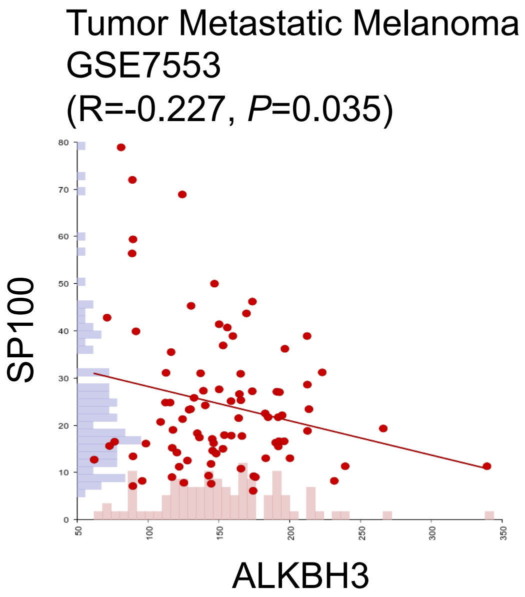

Supplementary Figure S6. The expression of SP100 was negatively associated with the expression of ALKBH3. Correlation analysis of ALKBH3 expression and SP100 expression in a cohort of metastatic melanoma samples (n=87). Significance was determined by *Pearson* correlation analysis (R = -0.227, *P* = 0.035).

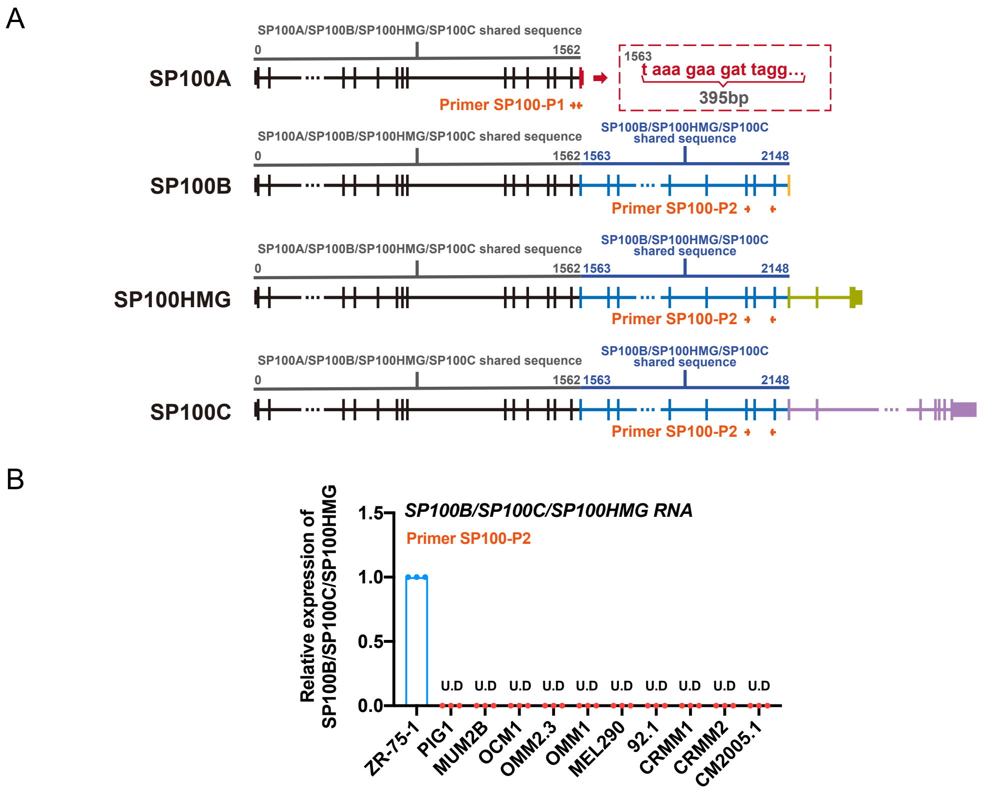

Supplementary Figure S7. The RNA expression of SP100B/SP100C/SP100HMG in ocular melanoma and normal melanocytes. (**A**) Sequence of SP100 isoforms. (**B**) qPCR data showing the SP100B/SP100C/SP100HMG expression in ocular melanoma cells, PIG1 cells relative to ZR-75–1 cell. The data are presented as the mean ± SD of experimental triplicates. U.D: undetected.

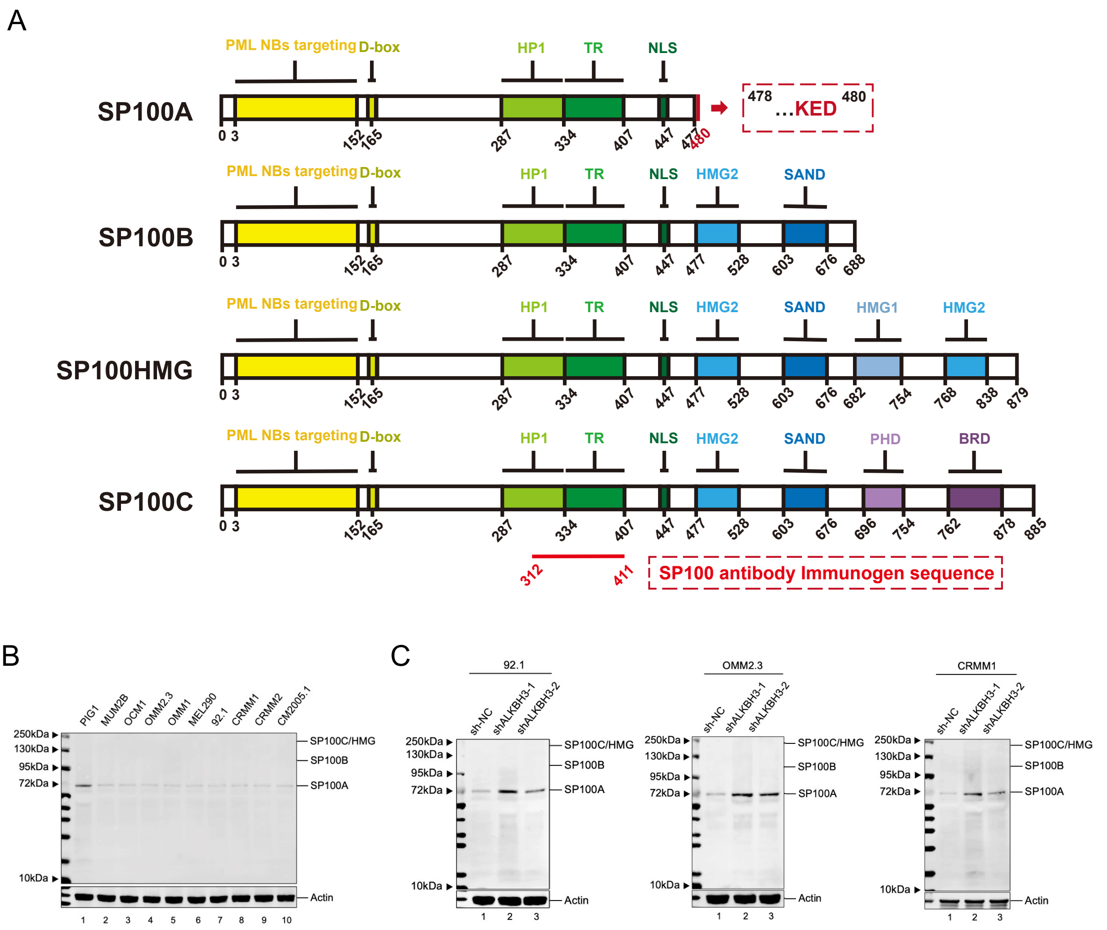

Supplementary Figure S8. The protein expression of SP100A, SP100B, SP100C and SP100HMG in ocular melanoma and normal melanocytes. (**A**) Protein domains of SP100 isoforms. D-box: Destruction-box (D-box); TR: trans-activating region; HMG: high mobility group; BRD: bromodomain; PHD: plant homeodomain. (**B**) Western blot showing SP100A, SP100B, SP100C and SP100HMG expression relative to ACTB expression in ocular melanoma cells (MUM2B, OCM1, OMM2.3, OMM1, MEL290, 92.1, CRMM1, CRMM2, CM2005.1) and normal pigmented cells (PIG1). The data are representative of experimental triplicates. (**C**) Western blot showing SP100A, SP100B, SP100C and SP100HMG expression relative to ACTB expression in wild-type and ALKBH3-deficient ocular melanoma cells (92.1, OMM2.3 and CRMM1).

A

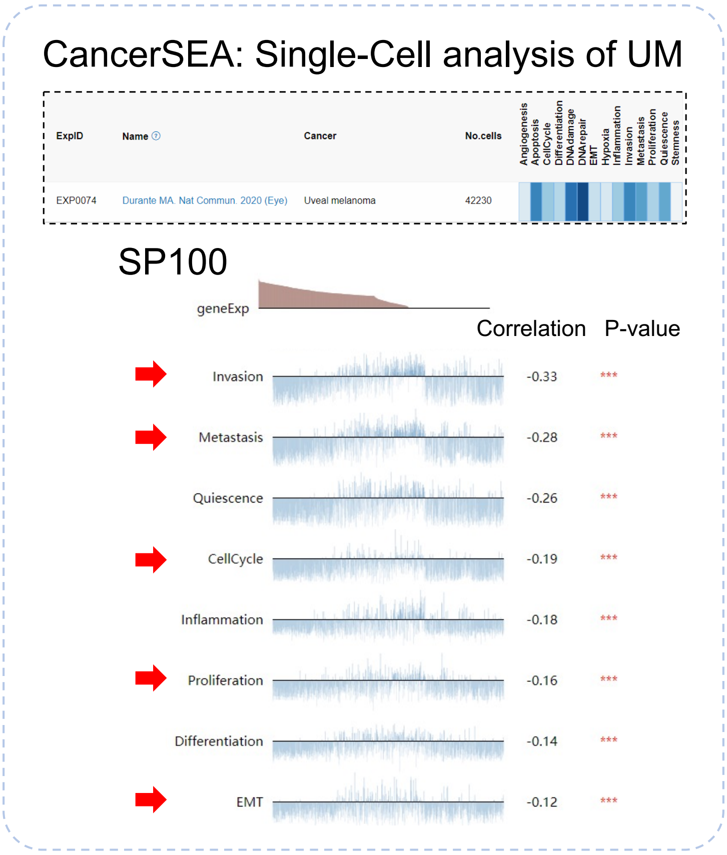

B

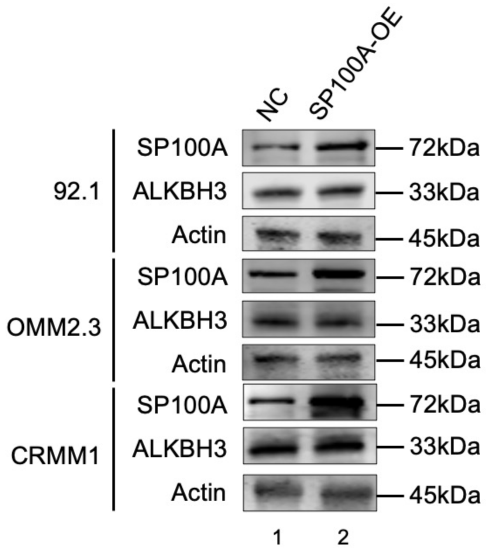

Supplementary Figure S9. SP100 prevents various oncogenic events in ocular melanoma. (**A**) Single-cell analysis data for ocular melanoma samples showing the correlation of SP100 expression and cancer-activation hallmarks using the CancerSEA platform. (**B**) Western blot showing SP100A and ALKBH3 expression relative to ACTB expression in ocular melanoma cells (92.1, OMM2.3 and CRMM1) upon SP100A overexpression. The data are representative of experimental triplicates.

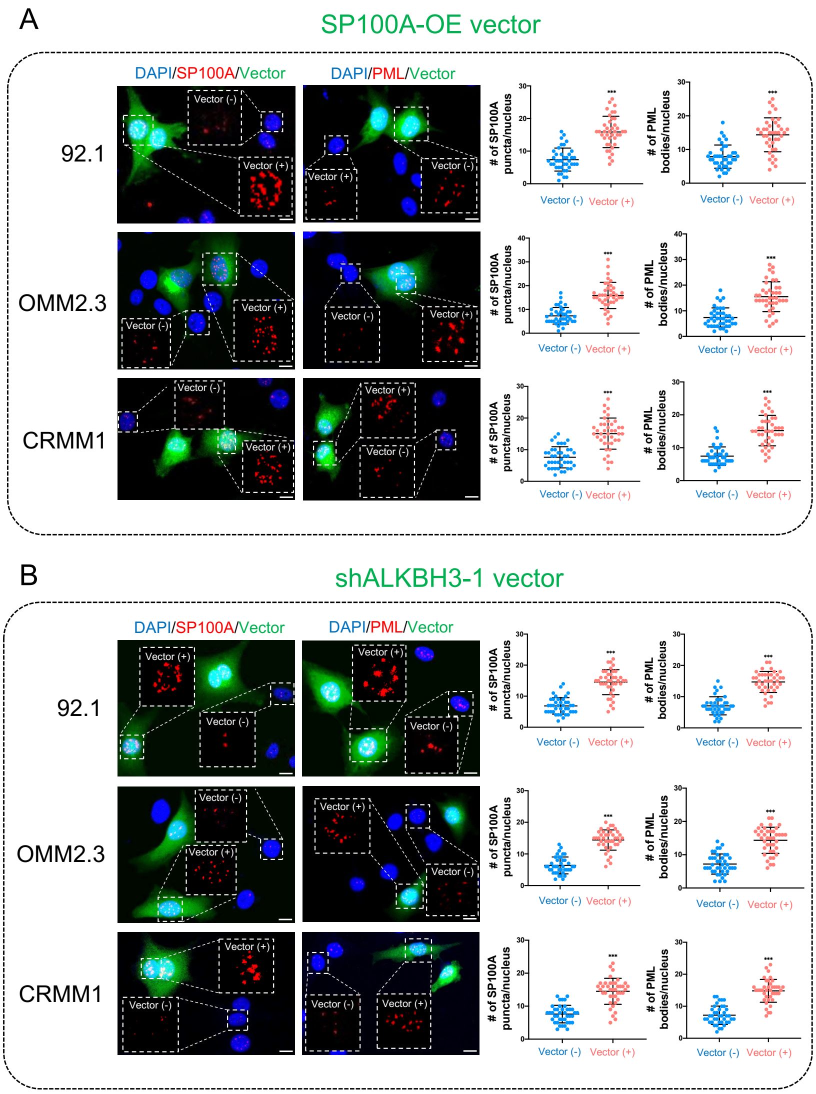

Supplementary Figure S10. Immunofluorescence analysis of the PML protein level through overexpressing SP100A and silencing of ALKBH3. (**A**) Immunofluorescence of Vector (green), SP100A (red), PML (red), and DAPI (blue) in ocular melanoma cells (92.1, OMM2.3 and CRMM1) upon SP100A overexpression. Scale bars: 10 μm. Number of SP100A puncta and PML bodies per nucleus (n = 40 cells) was quantified. Significance was determined by unpaired two-tailed Student's t test. *****P* < 0.0001. (**B**) Immunofluorescence of Vector (green), SP100A (red), PML (red), and DAPI (blue) in ocular melanoma cells (92.1, OMM2.3 and CRMM1) upon ALKBH3 knockdown. Scale bars: 10 μm. Number of SP100A puncta and PML bodies per nucleus (n = 40 cells) was quantified. Significance was determined by unpaired two-tailed Student's t test. *****P* < 0.0001.

A

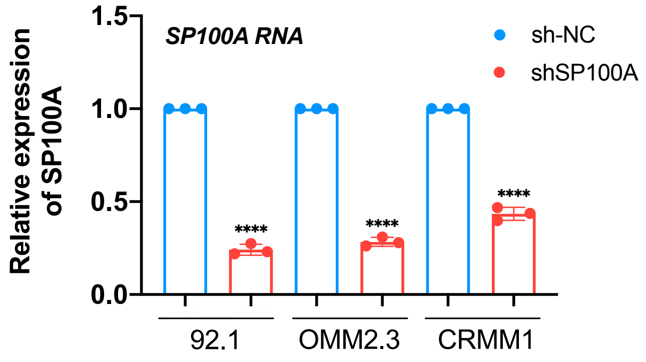

B

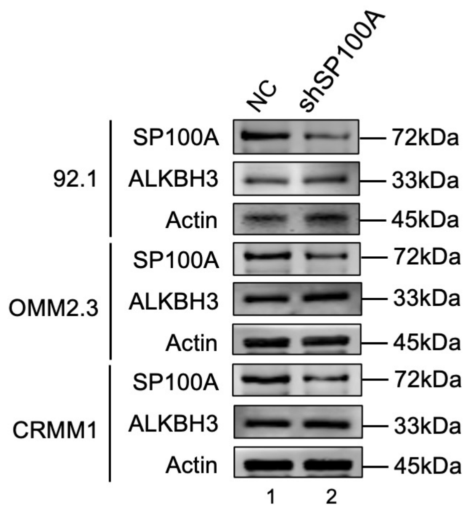

Supplementary Figure S11. SP100A presented with decreased expression in SP100A-deficient cells. (**A**) qPCR data showing the SP100A expression in ocular melanoma cells (92.1, OMM2.3 and CRMM1) upon SP100A knockdown. The data are presented as the mean ± SD of experimental triplicates. Significance was determined by unpaired two-tailed Student's t test. ****$P < 0.0001$. (**B**) Western blot of SP100A and ALKBH3 relative to ACTB in ocular melanoma cells (92.1, OMM2.3 and CRMM1) upon SP100A knockdown.

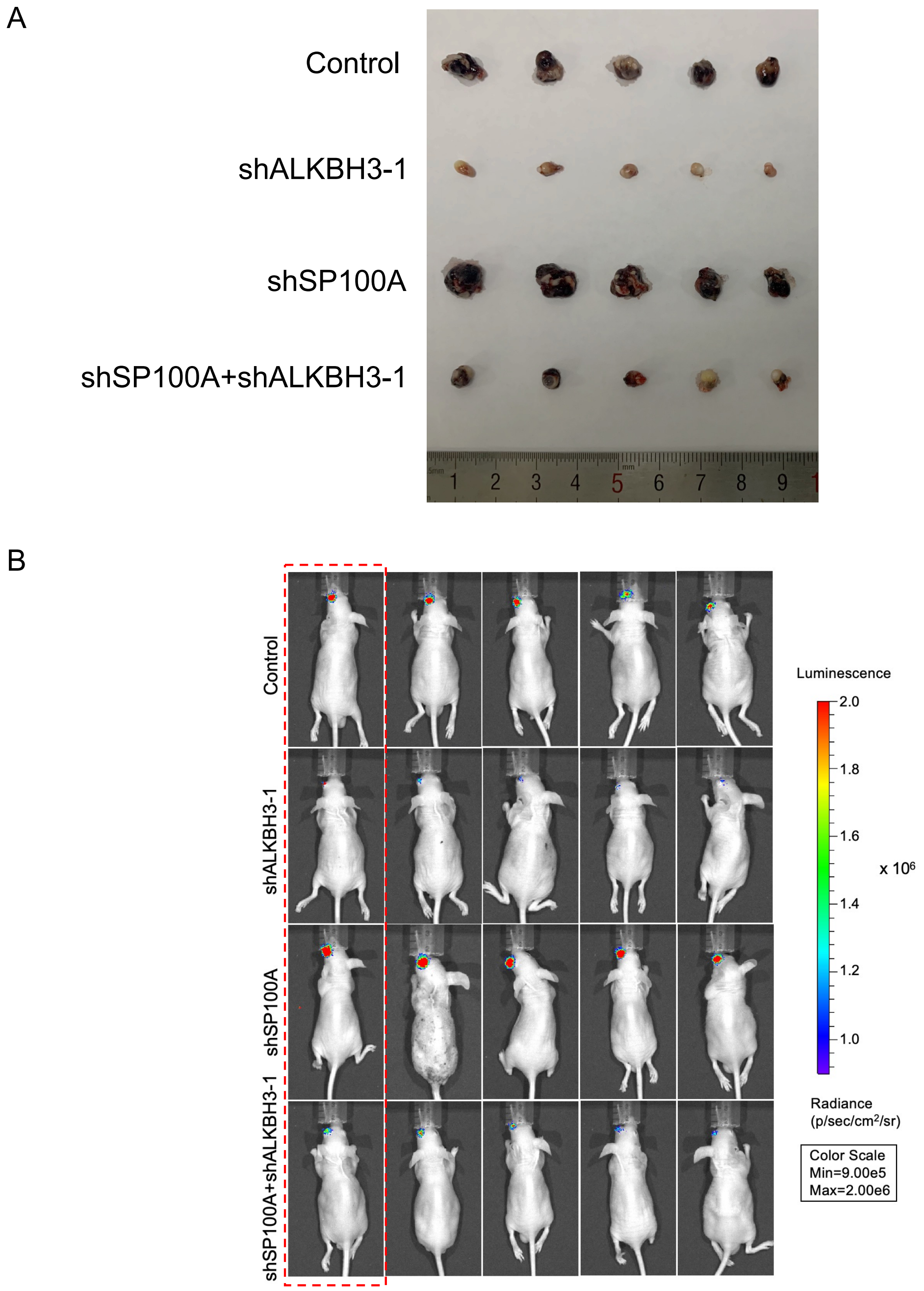

Supplementary Figure S12. The anticancer effects of ALKBH3 knockdown were partially blocked by SP100A silencing in vivo. (**A**) Images showing suppression of the growth of orthotopic xenografts tumors derived from ALKBH3-deficient 92.1 cells upon SP100 silencing. The data were obtained from five experimental replicates. (**B**) Images acquired with the small animal imaging system showing suppression of bioluminescent signals in orthotopic xenografts derived from ALKBH3-deficient 92.1 cells upon SP100A silencing. The data were obtained from five experimental replicates.

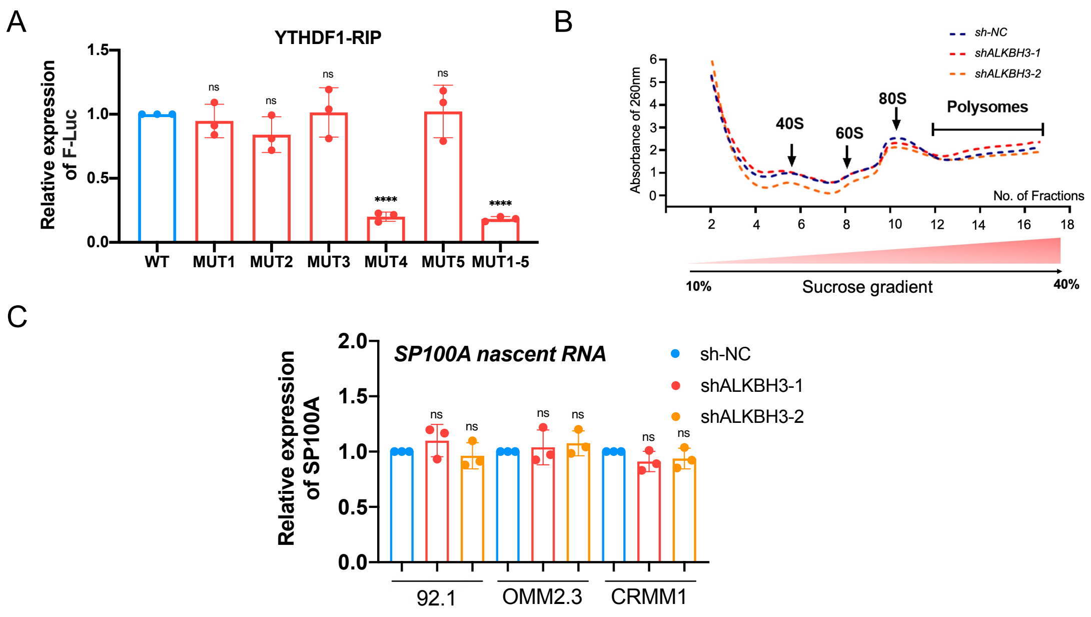

Supplementary Figure S13. Analysis of RNA fate of SP100A. (**A**) RIP-qPCR assay demonstrated the relative F-luc expression of the wild-type and six mutant SP100A 5'UTR reporter vectors by YTHDF1. The data are presented as the mean ± SD of experimental triplicates. Significance was determined by unpaired two-tailed Student's *t* test. ****P < 0.0001. (**B**) Polysome profiling assays upon ALKBH3 knockdown. Fractionation of lysates from 92.1 cells with or without ALKBH3 knockdown. Significance was determined by unpaired two-tailed Student's *t* test. ***P < 0.001, ****P < 0.0001. (**C**) qPCR data showing nascent SP100A RNA expression in ocular melanoma cells (92.1, OMM2.3 and CRMM1) upon ALKBH3 knockdown. The data are presented as the mean ± SD of experimental triplicates. Significance was determined by unpaired two-tailed Student's t test.

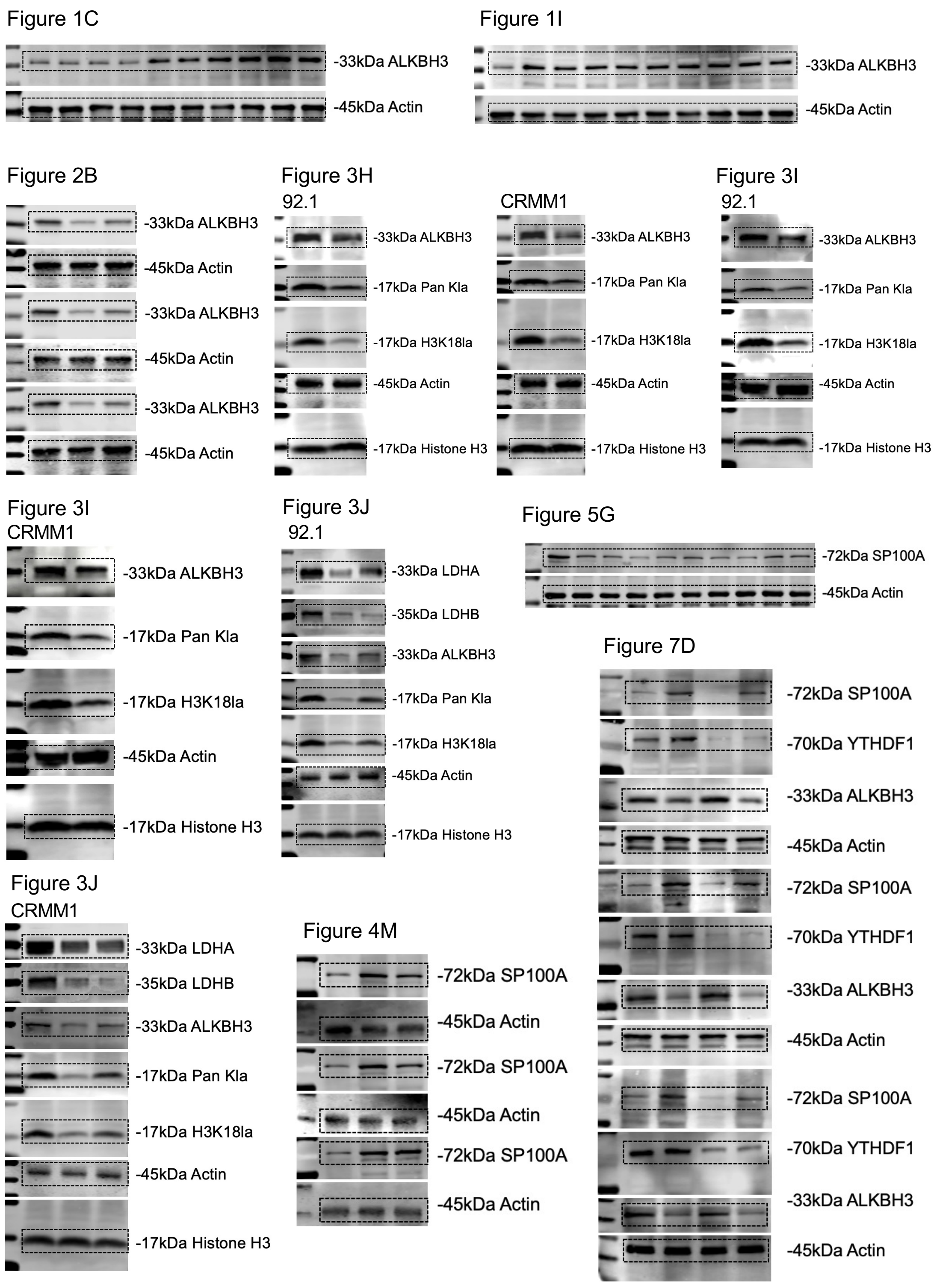

Supplementary Figure S14. Unprocessed Western blot.

Figure 5M

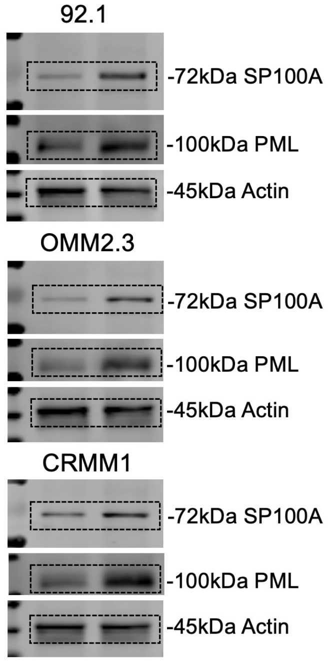

Figure 6F

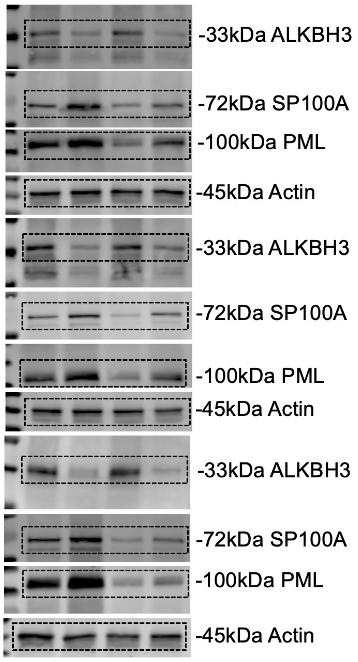

Supplementary Figure S9B

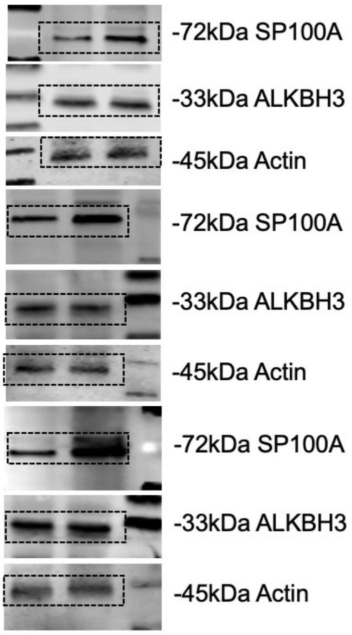

Supplementary Figure S11B

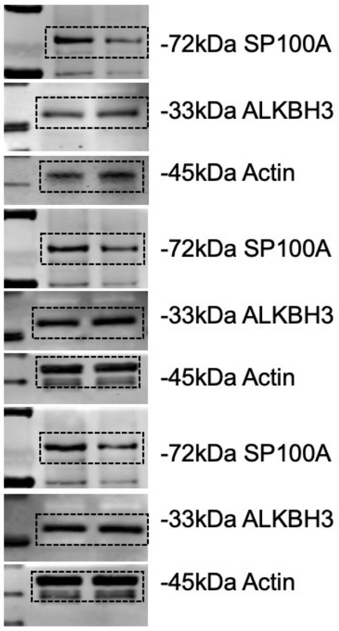

Supplementary Figure S15. Unprocessed Western blot.

Supplementary Table S1. Primers and shRNAs sequences used in this study.

Primer name	Sequence (5'-3')	Purpose
ALKBH3-F	GCCACGAGTGATTGACAGAG	PCR
ALKBH3-R	ATGCGGTTCTTTAGTGTGCG	PCR
SP100-General-F	TAGGGTGGGAAGATGGCAGG	PCR
SP100-General-R	TGGTCTTCCGTGAACATCCTTT	PCR
SP100-P1-F	AAGCATGGTGAGAAGGCTCC	PCR
SP100-P1-R	CAAGTCTTGGCATCCTAATCTTCT	PCR
SP100-P2-F	GAAGAAAAGCCAACACTAGACCT	PCR
SP100-P2-R	ACACTTCTTTGAGGTTCCTTGT	PCR
YTHDF1-F	CGTGGACACCCAGAGAACAA	PCR
YTHDF1-R	TGCCCAAAAACAGCATCGTG	PCR
Fluc-F	CACCTTCGTGACTTCCCATT	PCR
Fluc-R	TGACTGAATCGGACACAAGC	PCR
ACTB-F	GATTCCTATGTGGGCGACGA	PCR
ACTB-R	AGGTCTCAAACATGATCTGGGT	PCR
a site-F	ACCTGGTAATTCAGCCCACAG	PCR
a site-R	CCTCTCAGCTGGCTCTTTAGT	PCR
b site-F	GATGTGGGACTACCCGGACT	PCR
b site-R	TTCCTCTCGACGTTACGCAG	PCR
c site-F	TTGCTGGAGTCGGTGCTTCA	PCR
c site-R	GCACCCAGCAGTAAGTCAGG	PCR
d site-F	TCCAAAGTTGGTGAGAGATGCT	PCR
d site-R	AGCCAATCTGCAAGCAGTCA	PCR
ALKBH3-shRNA1		shRNA
Sence	GGAACAGCTTTGTCAAGAT	
Antisence	ATCTTGACAAAGCTGTTCC	
ALKBH3-shRNA2		shRNA
Sence	GCACATTTGAGATGAGAAA	
Antisence	TTTCTCATCTCAAATGTGC	
SP100-shRNA		shRNA
Sence	TGCGACTGGGTGATAAA	
Antisence	TTTATCACCCAGTCGCA	
YTHDF1-shRNA		shRNA
Sence	GTTCGTTACATCAGAAGGATA	
Antisence	TATCCTTCTGATGTAACGAAC	

Supplementary Table S2. Oligonucleotides used for luciferase reporter gene.

Oligonucleotides name	Sequence (5'-3')
SP100-5'UTR-WT	GCTTGGGGCC TGGGCAGCCA CACTGCACGC AGGCTGGGCC GACTGAGGGG CTCAGAGGCC AGGCTCTGAG GCCCACGCAG GGCCTAGGGT GGGAAG
SP100-5'UTR-MUT1	GCTTGGGGCC TGGGCAGCCA CACTGCACGC AGGCTGGGCC GACTGTGGGG CTCAGAGGCC AGGCTCTGAG GCCCACGCAG GGCCTAGGGT GGGAAG
SP100-5'UTR-MUT2	GCTTGGGGCC TGGGCAGCCA CACTGCACGC AGGCTGGGCC GACTGAGGGG CTCAGTGGCC AGGCTCTGAG GCCCACGCAG GGCCTAGGGT GGGAAG
SP100-5'UTR-MUT3	GCTTGGGGCC TGGGCAGCCA CACTGCACGC AGGCTGGGCC GACTGAGGGG CTCAGAGGCC AGGCTCTGTG GCCCACGCAG GGCCTAGGGT GGGAAG
SP100-5'UTR-MUT4	GCTTGGGGCC TGGGCAGCCA CACTGCACGC AGGCTGGGCC GACTGAGGGG CTCAGAGGCC AGGCTCTGAG GCCCACGCAG GGCCTAGGGT GGGTAG
SP100-5'UTR-MUT5	GCTTGGGGCC TGGGCAGCCA CACTGCACGC AGGCTGGGCC GACTGAGGGG CTCAGAGGCC AGGCTCTGAG GCCCACGCAG GGCCTAGGGT GGGATG
SP100-5'UTR-MUT1-5	GCTTGGGGCC TGGGCAGCCA CACTGCACGC AGGCTGGGCC GACTGTGGGG CTCAGTGGCC AGGCTCTGTG GCCCACGCAG GGCCTAGGGT GGGTTG

Supplementary Table S3. The differentially expressed proteins in proteomic analysis.

Accession	Protein Name	Gene Name	SH-1/REF	SH-2/REF	NC-1/REF	NC-2/REF	SH/NC	Significance
Q969S2	Endonuclease 8-like 2	NEIL2	1.912	2.117	0.697	0.641	3.012	8.8506E-66
Q10589	Bone marrow stromal antigen 2	BST2	1.829	1.845	0.646	0.653	2.828	1.0111E-58
Q9UID6	Zinc finger protein 639	ZNF639	1.898	1.760	0.683	0.653	2.739	2.9252E-55
Q8NHV4	Protein NEDD1	NEDD1	2.007	1.620	0.640	0.722	2.663	2.6016E-52
Q8TCB0	Interferon-induced protein 44	IFI44	1.642	1.788	0.730	0.609	2.562	2.0799E-48
O43462	Membrane-bound transcription factor site-2 protease	MBTPS2	1.675	1.793	0.657	0.743	2.478	3.5979E-45
O15162	Phospholipid scramblase 1	PLSCR1	1.748	1.535	0.637	0.716	2.427	3.3295E-43
P29728	2'-5'-oligoadenylate synthase 2	OAS2	1.525	1.533	0.699	0.682	2.215	4.1069E-35
P09914	Interferon-induced protein with tetratricopeptide repeats 1	IFIT1	1.565	1.572	0.742	0.715	2.153	8.9941E-33
O95786	Antiviral innate immune response receptor RIG-I	DDX58	1.551	1.557	0.740	0.759	2.073	8.2424E-30
O43805	Sjoegren syndrome nuclear autoantigen 1	SSNA1	1.538	1.620	0.827	0.781	1.964	8.5006E-26
Q53G44	Interferon-induced protein 44-like	IFI44L	1.442	1.416	0.741	0.780	1.880	9.4151E-23
Q9H8Y5	Ankyrin repeat and zinc finger domain-containing protein 1	ANKZF1	1.569	0.491	0.701	0.403	1.866	2.9177E-22
P20592	Interferon-induced GTP-binding protein Mx2	MX2	1.454	1.399	0.778	0.764	1.851	9.8016E-22
O14879	Interferon-induced protein with tetratricopeptide repeats 3	IFIT3	1.407	1.420	0.750	0.798	1.826	7.2535E-21
Q9Y646	Carboxypeptidase Q	CPQ	1.383	1.421	0.765	0.778	1.817	1.4985E-20
Q14703	Membrane-bound transcription factor site-1 protease	MBTPS1	1.588	1.410	0.816	0.847	1.802	5.1234E-20
Q8IVU3	Probable E3 ubiquitin-protein ligase HERC6	HERC6	1.295	1.568	0.728	0.879	1.781	2.5609E-19
P42224	Signal transducer and activator of transcription 1-alpha/beta	STAT1	1.396	1.373	0.757	0.800	1.778	3.3287E-19
P13164	Interferon-induced transmembrane protein 1	IFITM1	1.440	1.366	0.786	0.811	1.757	1.7587E-18
P01893	Putative HLA class I histocompatibility antigen, alpha chain H	HLA-H	1.632	1.270	0.814	0.864	1.731	1.3803E-17
Q9H3M7	Thioredoxin-interacting protein	TXNIP	1.288	1.219	0.756	0.705	1.716	4.3601E-17
Q01650	Large neutral amino acids transporter small subunit 1	SLC7A5	1.396	1.378	0.785	0.838	1.709	7.2459E-17
Q8N1Q1	Carbonic anhydrase 13	CA13	1.458	1.376	0.874	0.808	1.686	4.3805E-16
Q9NUL5	Shiftless antiviral inhibitor of ribosomal frameshifting protein	SHFL	1.321	1.395	0.756	0.860	1.681	6.256E-16
Q9Y5U4	Insulin-induced gene 2 protein	INSIG2	1.623	0.786	0.919	0.592	1.594	3.7021E-13
O14933	Ubiquitin/ISG15-conjugating enzyme E2 L6	UBE2L6	1.338	1.290	0.816	0.834	1.593	4.0755E-13
Q9Y6K5	2'-5'-oligoadenylate synthase 3	OAS3	1.343	1.299	0.830	0.829	1.592	4.45E-13
P05161	Ubiquitin-like protein ISG15	ISG15	1.342	1.246	0.816	0.818	1.583	8.6504E-13
P07305	Histone H1.0	H1-0	1.363	1.326	0.859	0.864	1.561	4.0801E-12
Q9P2W9	Syntaxin-18	STX18	1.304	1.416	0.882	0.865	1.557	5.1985E-12
Q9Y3Z3	Deoxynucleoside triphosphate triphosphohydrolase SAMHD1	SAMHD1	1.323	1.309	0.860	0.830	1.556	5.5197E-12
P52630	Signal transducer and activator of transcription 2	STAT2	1.316	1.299	0.835	0.849	1.552	7.3362E-12
P10071	Transcriptional activator GLI3	GLI3	1.317	0.898	0.864	0.589	1.525	5.0324E-11
Q9UDT6	CAP-Gly domain-containing linker protein 2	CLIP2	1.385	1.181	0.873	0.828	1.509	1.4185E-10
P22694	cAMP-dependent protein kinase catalytic subunit beta	PRKACB	1.324	1.350	0.929	0.867	1.488	5.7645E-10

A6NEE1	Pleckstrin homology domain-containing family D member 1	PLEKHD1	1.331	1.287	0.886	0.883	1.480	1.0052E-09
P27701	CD82 antigen	CD82	1.296	1.334	0.868	0.912	1.477	1.2086E-09
O00182	Galectin-9	LGALS9	1.299	1.244	0.845	0.887	1.469	2.0983E-09
Q9BYC8	39S ribosomal protein L32, mitochondrial	MRPL32	1.284	1.232	0.840	0.874	1.468	2.2495E-09
P23497	Nuclear autoantigen Sp-100	SP100	1.193	1.214	0.790	0.864	1.455	5.1131E-09
Q5K651	Sterile alpha motif domain-containing protein 9	SAMD9	1.242	1.278	0.885	0.859	1.445	9.7342E-09
Q53GL7	Protein mono-ADP-ribosyltransferase PARP10	PARP10	1.236	1.247	0.876	0.853	1.437	1.6012E-08
Q9H930	Nuclear body protein SP140-like protein	SP140L	1.111	1.196	0.771	0.850	1.423	3.7864E-08
Q96HD1	Protein disulfide isomerase CRELD1	CRELD1	1.269	1.181	0.839	0.895	1.412	7.3595E-08
Q9NZT2	Opioid growth factor receptor	OGFR	1.327	1.282	0.997	0.850	1.412	7.4107E-08
P01889	HLA class I histocompatibility antigen, B alpha chain	HLA-B	1.201	1.232	0.848	0.875	1.412	7.5318E-08
Q460N5	Protein mono-ADP-ribosyltransferase PARP14	PARP14	1.279	1.217	0.923	0.857	1.402	1.3783E-07
P39060	Collagen alpha-1(XVIII) chain	COL18A1	1.274	1.200	0.872	0.904	1.393	2.3212E-07
O14787	Transportin-2	TNPO2	1.211	1.282	0.852	0.951	1.383	4.2328E-07
P13637	Sodium/potassium-transporting ATPase subunit alpha-3	ATP1A3	1.200	1.242	0.858	0.913	1.379	5.3381E-07
P24385	G1/S-specific cyclin-D1	CCND1	0.939	1.730	0.652	1.285	1.378	5.7573E-07
Q8IVG5	Sterile alpha motif domain-containing protein 9-like	SAMD9L	1.153	1.244	0.831	0.913	1.375	6.9157E-07
P19525	Interferon-induced, double-stranded RNA-activated protein kinase	EIF2AK2	1.216	1.201	0.870	0.891	1.373	7.5664E-07
P55283	Cadherin-4	CDH4	1.190	1.362	0.845	1.017	1.370	8.8992E-07
Q6PJF5	Inactive rhomboid protein 2	RHBDF2	1.001	1.015	0.688	0.784	1.369	9.3505E-07
Q4L180	Filamin A-interacting protein 1-like	FILIP1L	1.231	1.402	0.828	1.109	1.360	1.6128E-06
Q9Y6D5	Brefeldin A-inhibited guanine nucleotide-exchange protein 2	ARFGEF2	1.298	1.143	0.973	0.828	1.355	2.1178E-06
Q5H9U9	Probable ATP-dependent RNA helicase DDX60-like	DDX60L	1.184	1.182	0.865	0.883	1.354	2.2198E-06
P09913	Interferon-induced protein with tetratricopeptide repeats 2	IFIT2	1.207	1.224	0.893	0.903	1.354	2.2735E-06
Q9UQ13	Leucine-rich repeat protein SHOC-2	SHOC2	1.210	1.100	0.833	0.883	1.347	3.356E-06
O00469	Procollagen-lysine,2-oxoglutarate 5-dioxygenase 2	PLOD2	1.167	1.152	0.863	0.881	1.330	8.5902E-06
Q06413	Myocyte-specific enhancer factor 2C	MEF2C	1.157	1.118	0.920	0.792	1.329	8.8432E-06
O15460	Prolyl 4-hydroxylase subunit alpha-2	P4HA2	1.140	1.125	0.854	0.852	1.327	9.9144E-06
P13726	Tissue factor	F3	1.053	1.122	0.778	0.861	1.327	1.0143E-05
O43294	Transforming growth factor beta-1-induced transcript 1 protein	TGFB1I1	1.168	1.077	0.830	0.865	1.325	1.1173E-05
Q5HYK7	SH3 domain-containing protein 19	SH3D19	1.096	1.151	0.861	0.842	1.319	1.5167E-05
Q16666	Gamma-interferon-inducible protein 16	IFI16	1.210	1.172	0.911	0.902	1.314	2.0183E-05
Q92522	Histone H1.10	H1-10	1.162	1.146	0.849	0.909	1.313	2.144E-05
Q8IXQ6	Protein mono-ADP-ribosyltransferase PARP9	PARP9	1.223	1.150	0.856	0.952	1.313	2.1667E-05
Q8IY21	Probable ATP-dependent RNA helicase DDX60	DDX60	1.222	1.197	0.930	0.914	1.312	2.2275E-05
Q96A26	Protein FAM162A	FAM162A	1.128	1.118	0.841	0.873	1.310	2.4458E-05

P28838	Cytosol aminopeptidase	LAP3	1.170	1.199	0.913	0.905	1.303	3.5315E-05
P22415	Upstream stimulatory factor 1	USF1	0.810	1.593	0.883	0.973	1.295	5.4693E-05
Q86YZ3	Hornerin	HRNR	1.531	0.871	1.268	0.588	1.294	5.5788E-05
Q8IYM9	E3 ubiquitin-protein ligase TRIM22	TRIM22	1.204	1.192	0.927	0.925	1.293	5.8238E-05
Q16877	6-phosphofructo-2-kinase/fructose-2,6-bisphosphatase 4	PFKFB4	1.101	1.115	0.865	0.850	1.292	6.2261E-05
P09543	2',3'-cyclic-nucleotide 3'-phosphodiesterase	CNP	1.191	1.185	0.925	0.916	1.291	6.6511E-05
Q9HB58	Sp110 nuclear body protein	SP110	1.154	1.142	0.847	0.938	1.286	8.5215E-05
P32455	Guanylate-binding protein 1	GBP1	1.204	1.170	0.929	0.920	1.284	9.1488E-05
Q9ULC8	Palmitoyltransferase ZDHHC8	ZDHHC8	1.226	1.193	0.884	1.007	1.279	0.00011757
P10321	HLA class I histocompatibility antigen, C alpha chain	HLA-C	1.207	1.158	0.903	0.946	1.279	0.00012131
Q13077	TNF receptor-associated factor 1	TRAF1	1.172	1.183	0.918	0.930	1.275	0.00014625
P61769	Beta-2-microglobulin	B2M	1.166	1.172	0.909	0.929	1.272	0.00017069
Q00653	Nuclear factor NF-kappa-B p100 subunit	NFKB2	1.251	1.155	0.970	0.922	1.272	0.00017249
Q13207	T-box transcription factor TBX2	TBX2	1.029	1.056	0.791	0.850	1.271	0.00017667
Q08380	Galectin-3-binding protein	LGALS3BP	1.159	1.137	0.905	0.904	1.269	0.00019502
P13674	Prolyl 4-hydroxylase subunit alpha-1	P4HA1	1.133	1.118	0.906	0.868	1.269	0.00019965
Q8IVF4	Dynein heavy chain 10, axonemal	DNAH10	1.124	1.094	0.808	0.941	1.268	0.00020782
Q8TDB6	E3 ubiquitin-protein ligase DTX3L	DTX3L	1.146	1.141	0.884	0.924	1.264	0.00024461
P32321	Deoxycytidylate deaminase	DCTD	1.163	1.522	1.062	1.065	1.263	0.00026691
P49840	Glycogen synthase kinase-3 alpha	GSK3A	1.059	1.527	1.020	1.029	1.263	0.00026729
Q6KB66	Keratin, type II cytoskeletal 80	KRT80	1.140	1.118	0.891	0.901	1.261	0.00028757
Q08AF3	Schlafen family member 5	SLFN5	1.185	1.144	0.920	0.927	1.261	0.00029293
Q00403	Transcription initiation factor IIB	GTF2B	1.250	1.161	1.021	0.892	1.260	0.00029754
P27797	Calreticulin	CALR	1.143	1.119	0.891	0.908	1.258	0.00033621
Q96BY9	Store-operated calcium entry-associated regulatory factor	SARAF	1.129	1.281	0.892	1.026	1.256	0.00035886
P51808	Dynein light chain Tctex-type 3	DYNLT3	1.209	1.037	0.921	0.868	1.256	0.0003655
Q96BI3	Gamma-secretase subunit APH-1A	APH1A	1.089	1.141	0.860	0.917	1.255	0.00037835
P50895	Basal cell adhesion molecule	BCAM	1.043	1.060	0.816	0.862	1.253	0.0004198
Q9BTM9	Ubiquitin-related modifier 1	URM1	1.090	1.125	0.865	0.903	1.252	0.00042988
P19474	E3 ubiquitin-protein ligase TRIM21	TRIM21	1.188	1.188	0.983	0.916	1.251	0.00046625
Q9Y5W8	Sorting nexin-13	SNX13	1.121	1.127	0.866	0.932	1.250	0.00047661
P62875	DNA-directed RNA polymerases I, II, and III subunit RPABC5	POLR2L	1.216	1.860	1.322	1.141	1.249	0.0005112
P78357	Contactin-associated protein 1	CNTNAP1	1.039	1.037	0.802	0.864	1.246	0.00059022
Q9BYM8	RanBP-type and C3HC4-type zinc finger-containing protein 1	RBCK1	1.083	1.150	0.879	0.915	1.245	0.00061451
P51787	Potassium voltage-gated channel subfamily KQT member 1	KCNQ1	1.154	1.043	0.850	0.916	1.244	0.00063286
Q16875	6-phosphofructo-2-kinase/fructose-2,6-bisphosphatase 3	PFKFB3	1.064	1.054	0.822	0.881	1.243	0.00066642
Q07002	Cyclin-dependent kinase 18	CDK18	1.010	1.091	0.838	0.852	1.242	0.00068446
Q9NUP1	Biogenesis of lysosome-related organelles complex 1 subunit 4	BLOC1S4	1.126	1.277	0.982	0.957	1.240	0.00078001
Q9UL42	Paraneoplastic antigen Ma2	PNMA2	1.115	1.158	0.895	0.939	1.239	0.00079992
Q6P2I3	Fumarylacetoacetate hydrolase domain-containing protein 2B	FAHD2B	1.155	1.148	0.939	0.919	1.239	0.00080612
Q8WV24	Pleckstrin homology-like domain family A member 1	PHLDA1	1.189	1.044	0.922	0.883	1.238	0.00085143

Q9P225	Dynein heavy chain 2, axonemal	DNAH2	1.114	1.044	0.843	0.901	1.237	0.00087615
Q9NWZ5	Uridine-cytidine kinase-like 1	UCKL1	1.025	1.243	0.854	0.982	1.236	0.0009357
Q8N5A5	Zinc finger CCCH-type with G patch domain-containing protein	ZGPAT	1.269	1.078	0.935	0.968	1.233	0.00103602
O15182	Centrin-3	CETN3	1.131	1.042	0.959	0.804	1.233	0.00104692
Q8TEP8	Centrosomal protein of 192 kDa	CEP192	1.104	1.056	0.828	0.925	1.232	0.00109299
Q86TB9	Protein PAT1 homolog 1	PATL1	1.115	1.254	0.945	0.980	1.231	0.00113932
P29373	Cellular retinoic acid-binding protein 2	CRABP2	1.083	1.085	0.903	0.860	1.229	0.00122557
P52746	Zinc finger protein 142	ZNF142	1.082	1.084	0.919	0.844	1.229	0.00126165
Q8WUJ0	Serine/threonine/tyrosine-interacting protein	STYX	1.140	1.109	0.899	0.931	1.229	0.00127397
Q06136	3-ketodihydrosphingosine reductase	KDSR	1.188	1.073	0.886	0.956	1.227	0.00137184
P11169	Solute carrier family 2, facilitated glucose transporter member 3	SLC2A3	1.083	1.139	0.885	0.927	1.226	0.00144317
P55210	Caspase-7	CASP7	1.178	1.109	0.984	0.883	1.225	0.00147334
P58335	Anthrax toxin receptor 2	ANTXR2	1.152	1.108	0.950	0.896	1.224	0.00152492
P00558	Phosphoglycerate kinase 1	PGK1	1.117	1.085	0.918	0.882	1.224	0.0015584
P00750	Tissue-type plasminogen activator	PLAT	1.148	1.168	0.983	0.910	1.224	0.00157773
Q96BP3	Peptidylprolyl isomerase domain and WD repeat-containing protein 1	PPWD1	1.149	1.107	0.967	0.882	1.220	0.00184299
Q9UBB9	Tuftelin-interacting protein 11	TFIP11	1.090	1.077	0.857	0.923	1.217	0.00206091
Q9BQE5	Apolipoprotein L2	APOL2	1.145	1.141	0.930	0.948	1.217	0.00207378
P35520	Cystathionine beta-synthase	CBS	1.024	1.147	0.769	1.015	1.217	0.00211945
P24390	ER lumen protein-retaining receptor 1	KDELR1	0.940	0.896	0.796	0.714	1.215	0.00224457
P04439	HLA class I histocompatibility antigen, A alpha chain	HLA-A	1.132	1.121	0.922	0.933	1.214	0.00237632
P14921	Protein C-ets-1	ETS1	0.992	1.171	0.859	0.923	1.213	0.00245321
Q92597	Protein NDRG1	NDRG1	1.093	0.959	0.862	0.830	1.213	0.00246538
Q6IQ26	DENN domain-containing protein 5A	DENND5A	1.118	1.087	0.905	0.913	1.213	0.00249125
Q13535	Serine/threonine-protein kinase ATR	ATR	1.008	1.028	0.826	0.854	1.212	0.00260054
Q12882	Dihydropyrimidine dehydrogenase [NADP(+)]	DPYD	1.060	1.058	0.842	0.909	1.210	0.00284516
Q9H5V8	CUB domain-containing protein 1	CDCP1	1.150	1.146	0.958	0.942	1.209	0.00299077
Q8IVW4	Cyclin-dependent kinase-like 3	CDKL3	1.112	1.139	0.973	0.892	1.207	0.00318547
Q06330	Recombining binding protein suppressor of hairless	RBPJ	1.201	1.030	0.954	0.897	1.205	0.00344695
Q00978	Interferon regulatory factor 9	IRF9	1.142	1.129	1.005	0.881	1.204	0.00359849
Q8NI22	Multiple coagulation factor deficiency protein 2	MCFD2	1.184	1.186	0.864	1.106	1.203	0.00377341
Q96CU9	FAD-dependent oxidoreductase domain-containing protein 1	FOXRED1	1.133	1.117	0.847	1.025	1.202	0.0039371
P06744	Glucose-6-phosphate isomerase	GPI	1.094	1.047	0.900	0.882	1.202	0.00395704
P00973	2'-5'-oligoadenylate synthase 1	OAS1	1.106	1.133	0.950	0.912	1.202	0.00396005
Q8TBP5	Membrane protein FAM174A	FAM174A	1.049	1.028	0.910	0.818	1.202	0.003984
Q9UL46	Proteasome activator complex subunit 2	PSME2	1.148	1.137	0.949	0.954	1.201	0.00410366
Q9BU89	Deoxyhypusine hydroxylase	DOHH	0.928	0.936	1.173	1.064	0.833	0.00189316
Q7Z6K5	Arpin	ARPIN	0.837	1.038	1.218	1.034	0.833	0.00185116
O43715	TP53-regulated inhibitor of apoptosis 1	TRIAP1	0.911	0.903	1.122	1.060	0.831	0.00162177

Q8NB15	Zinc finger protein 511	ZNF511	0.841	0.922	1.033	1.089	0.831	0.00161237
P20290	Transcription factor BTF3	BTF3	0.958	0.923	1.183	1.080	0.831	0.00160525
Q9UG56	Phosphatidylserine decarboxylase proenzyme, mitochondrial	PISD	0.839	0.895	1.050	1.042	0.829	0.0014276
P62304	Small nuclear ribonucleoprotein E	SNRPE	0.752	1.011	1.059	1.072	0.827	0.00123631
Q96GC9	Vacuole membrane protein 1	VMP1	1.048	0.791	1.104	1.120	0.827	0.00119801
Q6P582	Mitotic-spindle organizing protein 2A	MZT2A	0.821	0.928	1.097	1.022	0.825	0.00108134
P13928	Annexin A8	ANXA8	0.892	0.953	1.137	1.101	0.825	0.00103341
Q9NS87	Kinesin-like protein KIF15	KIF15	0.941	1.020	1.161	1.218	0.824	0.00099464
P30838	Aldehyde dehydrogenase, dimeric NADP-preferring	ALDH3A1	0.854	0.949	1.118	1.069	0.824	0.00098507
P05204	Non-histone chromosomal protein HMG-17	HMGN2	0.907	0.887	1.131	1.046	0.824	0.00098362
Q13765	Nascent polypeptide-associated complex subunit alpha	NACA	0.926	0.930	1.102	1.153	0.823	0.00090573
Q9H4L7	SWI/SNF-related matrix-associated actin-dependent regulator of chromatin subfamily A containing DEAD/H box 1	SMARCAD1	0.909	0.991	1.015	1.298	0.821	0.00080888
O14681	Etoposide-induced protein 2.4 homolog	EI24	0.931	0.980	1.128	1.200	0.821	0.00076053
Q86TS9	39S ribosomal protein L52, mitochondrial	MRPL52	0.944	0.882	1.236	0.991	0.820	0.00073702
Q99595	Mitochondrial import inner membrane translocase subunit Tim17-A	TIMM17A	0.883	0.906	1.161	1.021	0.820	0.00072773
Q8WV99	AN1-type zinc finger protein 2B	ZFAND2B	0.927	0.920	1.126	1.129	0.819	0.00066582
Q3B726	DNA-directed RNA polymerase I subunit RPA43	POLR1F	0.862	0.951	1.158	1.057	0.818	0.00064084
P05090	Apolipoprotein D	APOD	1.010	0.937	1.202	1.186	0.815	0.00050602
P59536	Taste receptor type 2 member 41	TAS2R41	0.767	0.884	0.939	1.087	0.815	0.00048377
P98179	RNA-binding protein 3	RBM3	0.787	0.985	1.067	1.114	0.813	0.00040779
O60613	Selenoprotein F	SELENOF	0.879	0.901	1.071	1.120	0.812	0.00039371
Q5JV73	FERM and PDZ domain-containing protein 3	FRMPD3	0.857	0.818	1.097	0.967	0.812	0.00038427
Q9NZP5	Olfactory receptor 5AC2	OR5AC2	0.843	0.837	1.033	1.038	0.812	0.0003755
Q9H1E3	Nuclear ubiquitous casein and cyclin-dependent kinase substrate 1	NUCKS1	0.935	0.790	1.048	1.084	0.809	0.00030398
Q96RS0	Trimethylguanosine synthase	TGS1	0.814	1.008	1.109	1.150	0.806	0.00024603
O75683	Surfeit locus protein 6	SURF6	0.828	0.954	1.135	1.075	0.806	0.00024345
P63302	Selenoprotein W	SELENOW	0.903	0.872	1.076	1.126	0.806	0.00023548
Q5TBB1	Ribonuclease H2 subunit B	RNASEH2B	0.877	0.925	1.115	1.128	0.804	0.00019192
P05120	Plasminogen activator inhibitor 2	SERPINB2	0.959	0.955	1.198	1.187	0.802	0.00017517
O15446	DNA-directed RNA polymerase I subunit RPA34	POLR1G	0.961	0.994	1.289	1.148	0.802	0.00017492
A4FU01	Myotubularin-related protein 11	MTMR11	0.796	1.057	1.140	1.172	0.801	0.00016125
Q9UI32	Glutaminase liver isoform, mitochondrial	GLS2	0.904	0.930	1.201	1.096	0.799	0.00012492
O94972	E3 ubiquitin-protein ligase TRIM37	TRIM37	0.784	0.841	0.952	1.086	0.797	0.00011324
Q14011	Cold-inducible RNA-binding protein	CIRBP	0.893	0.921	1.117	1.168	0.794	8.022E-05
P52926	High mobility group protein HMGI-C	HMGA2	0.984	0.911	1.205	1.189	0.792	6.7836E-05
Q9Y487	V-type proton ATPase 116 kDa subunit a2	ATP6V0A2	0.831	0.803	1.035	1.032	0.790	6.0331E-05

Q96JA4	Membrane-spanning 4-domains subfamily A member 14	MS4A14	0.798	0.788	1.002	1.006	0.790	5.6149E-05
Q8IWS0	PHD finger protein 6	PHF6	0.850	0.987	1.283	1.048	0.788	4.838E-05
Q6NUP7	Serine/threonine-protein phosphatase 4 regulatory subunit 4	PPP4R4	0.838	0.785	1.043	1.016	0.788	4.7917E-05
Q9Y2H5	Pleckstrin homology domain-containing family A member 6	PLEKHA6	0.851	0.867	1.087	1.095	0.787	4.5151E-05
Q8WTV0	Scavenger receptor class B member 1	SCARB1	0.732	1.059	1.172	1.115	0.783	2.9577E-05
O14569	Cytochrome b561 domain-containing protein 2	CYB561D2	0.840	0.863	1.116	1.065	0.781	2.4287E-05
Q96J84	Kin of IRRE-like protein 1	KIRREL1	0.791	0.940	1.344	0.878	0.779	2.05E-05
O60706	ATP-binding cassette sub-family C member 9	ABCC9	0.847	0.761	1.122	0.943	0.778	1.9117E-05
Q9UMS0	NFU1 iron-sulfur cluster scaffold homolog, mitochondrial	NFU1	0.900	0.934	1.274	1.091	0.776	1.461E-05
Q08623	Pseudouridine-5'-phosphatase	PUDP	1.009	0.929	1.115	1.395	0.772	1.0329E-05
Q99757	Thioredoxin, mitochondrial	TXN2	1.016	0.849	1.240	1.176	0.772	9.946E-06
Q5EBL4	RILP-like protein 1	RILPL1	0.874	0.966	1.222	1.178	0.766	5.5528E-06
Q6PK04	Coiled-coil domain-containing protein 137	CCDC137	0.847	0.800	1.152	1.009	0.762	3.5866E-06
Q9BV57	1,2-dihydroxy-3-keto-5-methylthiopentene dioxygenase	ADI1	0.848	0.845	1.147	1.089	0.757	2.0493E-06
P47895	Aldehyde dehydrogenase family 1 member A3	ALDH1A3	0.809	0.809	1.077	1.089	0.747	5.977E-07
Q8NC42	E3 ubiquitin-protein ligase RNF149	RNF149	0.778	0.798	1.064	1.128	0.719	1.6333E-08
Q9BV44	THUMP domain-containing protein 3	THUMPD3	0.772	0.855	1.096	1.178	0.716	1.0562E-08
Q8TCF1	AN1-type zinc finger protein 1	ZFAND1	0.709	0.884	1.098	1.158	0.706	2.6239E-09
P13611	Versican core protein	VCAN	0.803	0.915	1.430	1.009	0.705	2.1293E-09
Q68DU8	BTB/POZ domain-containing protein KCTD16	KCTD16	0.691	0.695	0.988	1.009	0.694	4.2849E-10
Q9UPY5	Cystine/glutamate transporter	SLC7A11	0.834	0.814	1.243	1.164	0.685	9.2888E-11
Q13322	Growth factor receptor-bound protein 10	GRB10	0.722	0.755	0.904	1.259	0.683	6.8451E-11
P07203	Glutathione peroxidase 1	GPX1	0.772	0.782	1.138	1.161	0.676	2.2474E-11
O94808	Glutamine--fructose-6-phosphate aminotransferase [isomerizing] 2	GFPT2	0.672	0.625	1.033	0.923	0.663	2.0665E-12
P02748	Complement component C9	C9	0.653	0.809	1.210	1.276	0.588	8.7947E-20
Q9H6N6	Putative uncharacterized protein MYH16	MYH16	0.904	0.942	0.693	2.556	0.568	3.5002E-22
O75629	Protein CREG1	CREG1	0.818	0.864	1.099	2.053	0.534	5.2491E-27
O75764	Transcription elongation factor A protein 3	TCEA3	0.468	0.507	1.279	1.378	0.367	2.352E-66

Supplementary Table S4. The clinical characteristics of ocular melanoma patient cohorts in tissue chip assay.

Patient No.	Gender (0=Female, 1=Male)	Age (years old)	Time for recurrence (month, 0=No recurrence)	Relative expression of ALKBH3	Relative expression of SP100A
1	1	59	2	0.3	0.5
2	1	59	2	6.2	1.3
3	1	45	/	7.5	0.8
4	1	45	/	1.2	3.3
5	1	57	/	2.0	4.2
6	1	54	36	1.2	1.3
7	1	54	6	2.3	1.1
8	1	76	0	0.5	0.4
9	0	54	0	2.8	1.8
10	1	46	6	5.4	0.5
11	1	57	24	2.3	0.9
12	1	57	24	0.6	0.9
13	1	67	/	2.8	1.1
14	1	59	2	2.0	0.3
15	1	59	2	0.8	0.2
16	0	89	0	1.1	2.3
17	0	89	0	0.9	1.5
18	1	52	/	2.9	1.0
19	1	37	/	3.3	1.1
20	1	82	0	2.1	1.4
21	1	82	0	1.3	2.8
22	1	48	60	0.7	0.3
23	0	29	0	0.4	0.3
24	0	49	11	3.1	0.6
25	0	49	11	5.3	0.2
26	1	35	34	0.8	1.4
27	1	35	34	1.0	1.2
28	1	47	0	1.8	2.5
29	0	60	/	2.4	1.1
30	1	64	/	1.6	0.8
31	1	52	2	6.6	0.2
32	0	77	19	5.8	0.9
33	0	45	/	7.9	1.0
34	0	42	18	1.9	1.0
35	1	55	12	4.2	1.2

36	1	65	/	4.9	2.4
37	1	61	3	3.1	0.2
38	1	31	13	1.2	0.9
39	1	32	/	2.0	1.4
40	0	66	/	2.5	1.6
41	0	69	/	2.1	1.5
42	0	31	3	2.3	1.1
43	1	13	/	2.2	1.5
44	1	64	/	1.6	0.7
45	0	67	0	1.7	3.8
46	1	23	/	2.5	0.9
47	1	23	/	2.3	0.9
48	1	52	0	2.0	2.3
49	1	12	/	1.4	0.8
50	1	73	13	0.6	0.2
51	0	52	/	2.5	0.5
52	1	31	13	3.3	1.1
53	1	78	0	0.4	1.8
54	0	42	18	4.1	0.9
55	1	66	20	1.5	0.5
56	1	58	0	7.4	0.9
57	0	82	/	1.4	2.6
58	1	80	/	1.9	1.0
59	1	73	/	2.0	0.7
60	1	60	9	1.9	0.7
61	0	51	12	3.4	0.8
62	0	58	/	2.4	1.7
63	1	52	2	7.4	0.2
64	1	73	13	4.8	1.1
65	1	81	0	3.2	1.3
66	1	20	/	1.7	0.9
67	1	47	16	3.2	1.3
68	0	77	19	4.6	1.3
69	1	54	2	1.9	0.3
70	1	63	32	1.2	0.4
71	1	38	8	5.2	0.9
72	1	38	8	3.4	0.1
73	1	70	23	1.3	0.8
74	1	70	23	1.2	0.3
75	1	18	6	2.4	0.1
76	1	18	6	0.6	0.3

77	1	76	0	1.5	1.4
78	1	75	0	1.6	1.5
79	0	53	5	6.1	0.3
80	1	74	0	5.7	0.7
81	1	49	0	0.8	0.6
82	1	67	0	0.6	0.4

Supplementary Table S5. The clinical characteristics of melanocytic nevus patient cohorts in tissue chip assay.

Patient No.	Gender (0=Female, 1=Male)	Age (years old)	Relative expression of ALKBH3	Relative expression of SP100A
1	1	64	0.3	1.3
2	0	48	0.8	0.8
3	0	29	0.8	1.6
4	0	29	0.4	1.4
5	1	31	0.3	2.6
6	0	25	0.3	1.5
7	1	32	1.0	0.7
8	0	65	0.9	5.5
9	0	61	0.5	1.3
10	1	55	0.4	0.8
11	0	30	0.4	4.2
12	1	50	0.3	1.3
13	1	67	0.3	5.1
14	1	40	0.4	4.3
15	0	31	0.4	0.4
16	0	54	0.4	1.9
17	0	60	0.7	1.2
18	1	62	0.7	1.4
19	0	45	0.4	3.3
20	0	62	1.6	1.6
21	0	63	0.9	1.2
22	0	1	0.8	0.9
23	0	8	2.9	1.0
24	0	29	1.3	3.9
25	0	29	0.7	5.9
26	1	3	2.0	0.6
27	0	26	0.8	1.1
28	1	14	0.9	2.3

Supplementary Table S6. The clinical characteristics of patients with high and low ALKBH3 levels.

Stratified by ALKBH3			
	high	low	p-value
n	29	29	
age (mean (SD))	54.1 (15.4)	59.2 (18.3)	0.256
sex = male (%)	20 (69.0)	24 (82.8)	0.220
T stage (%)			0.452
T1	1 (3.4)	1 (3.4)	
T2	10 (34.5)	13 (44.8)	
T3	18 (62.1)	15 (51.7)	

Supplementary Table S7. The clinical characteristics of patients with high and low SP100A levels.

Stratified by SP100A			
	high	low	p-value
n	29	29	
age (mean (SD))	58.3 (18.7)	55.1 (15.2)	0.481
sex = male (%)	21 (72.4)	23 (79.3)	0.539
T stage (%)			0.979
T1	2 (4.5)	0 (0)	
T2	10 (34.5)	13 (44.8)	
T3	17 (58.6)	16 (55.2)	
